# Revealing the Dynamic Nature of Amplitude Modulated Neural Entrainment With Holo-Hilbert Spectral Analysis

**DOI:** 10.3389/fnins.2021.673369

**Published:** 2021-08-05

**Authors:** Chi-Hung Juan, Kien Trong Nguyen, Wei-Kuang Liang, Andrew J. Quinn, Yen-Hsun Chen, Neil G. Muggleton, Jia-Rong Yeh, Mark W. Woolrich, Anna C. Nobre, Norden E. Huang

**Affiliations:** ^1^Institute of Cognitive Neuroscience, National Central University, Taoyuan City, Taiwan; ^2^Cognitive Intelligence and Precision Healthcare Center, National Central University, Taoyuan City, Taiwan; ^3^Department of Psychology, Kaohsiung Medical University, Kaohsiung City, Taiwan; ^4^Faculty of Electronics Engineering, Posts and Telecommunications Institute of Technology, Ho Chi Minh City, Vietnam; ^5^Oxford Centre for Human Brain Activity, University of Oxford, Oxford, United Kingdom; ^6^Wellcome Centre for Integrative Neuroimaging, University of Oxford, Oxford, United Kingdom; ^7^Department of Psychology, Goldsmiths, University of London, London, United Kingdom; ^8^Institute of Cognitive Neuroscience, University College London, London, United Kingdom; ^9^Department of Experimental Psychology, University of Oxford, Oxford, United Kingdom; ^10^Data Analysis and Application Laboratory, The First Institute of Oceanography, Qingdao, China

**Keywords:** the dynamic visual entrainment, Holo-Hilbert spectral analysis, cross-frequency coupling, steady-state visual evoked potential, phase-amplitude coupling

## Abstract

Patterns in external sensory stimuli can rapidly entrain neuronally generated oscillations observed in electrophysiological data. Here, we manipulated the temporal dynamics of visual stimuli with cross-frequency coupling (CFC) characteristics to generate steady-state visual evoked potentials (SSVEPs). Although CFC plays a pivotal role in neural communication, some cases reporting CFC may be false positives due to non-sinusoidal oscillations that can generate artificially inflated coupling values. Additionally, temporal characteristics of dynamic and non-linear neural oscillations cannot be fully derived with conventional Fourier-based analyses mainly due to trade off of temporal resolution for frequency precision. In an attempt to resolve these limitations of linear analytical methods, Holo-Hilbert Spectral Analysis (HHSA) was investigated as a potential approach for examination of non-linear and non-stationary CFC dynamics in this study. Results from both simulation and SSVEPs demonstrated that temporal dynamic and non-linear CFC features can be revealed with HHSA. Specifically, the results of simulation showed that the HHSA is less affected by the non-sinusoidal oscillation and showed possible cross frequency interactions embedded in the simulation without any *a priori* assumptions. In the SSVEPs, we found that the time-varying cross-frequency interaction and the bidirectional coupling between delta and alpha/beta bands can be observed using HHSA, confirming dynamic physiological signatures of neural entrainment related to cross-frequency coupling. These findings not only validate the efficacy of the HHSA in revealing the natural characteristics of signals, but also shed new light on further applications in analysis of brain electrophysiological data with the aim of understanding the functional roles of neuronal oscillation in various cognitive functions.

## Introduction

### Background

Neural activity related to human behaviors are prominently implemented in a dynamic and non-linear manner (Buzsáki and Mizuseki, 2014; Clarke et al., 2015). Various forms of neural oscillations may play a critical role in these processes (e.g., Buzsaki, 2006) and these are typically categorized into activity in different frequency bands (i.e., delta, theta, alpha, beta, gamma, and high gamma) and commonly reported for magneto/electroencephalograms (MEG and EEG) and local field potentials (LFP) (e.g., Cole and Voytek, 2017). Neural cross-frequency coupling (CFC) represents the interactions between two neuronal oscillations of different frequencies and can occur both within a neural area or as inter-area communication (Singer, 1999; Salinas and Sejnowski, 2001; Varela et al., 2001; Fries, 2005; Canolty et al., 2006; Jensen and Colgin, 2007; Roach and Mathalon, 2008; Canolty and Knight, 2010; Giraud and Poeppel, 2012; Siegel et al., 2012; Hsu et al., 2014; Lopes-dos-Santos et al., 2018; Cole and Voytek, 2019; Hanslmayr et al., 2019; Nguyen et al., 2019; Siebenhühner et al., 2020; Giehl et al., 2021; Liang et al., 2021). One of the most examined forms of CFC is phase-amplitude coupling (PAC), where the phase of a lower frequency oscillation modulates the amplitude of a high frequency oscillation (Canolty et al., 2006; Canolty and Knight, 2010). It has been suggested that PAC can not only be observed locally within the same signal in local field potentials in rats and in human intracranial EEG, but could also reflect the long-range interactions between regions (Jensen and Colgin, 2007; Nandi et al., 2019; Siebenhühner et al., 2020). For example, a seminal work by Canolty et al. (2006) found that the amplitude of gamma activity was coupled with the phase of theta oscillations in humans. However, although multiple competing algorithms and approaches have been proposed for conducting PAC analysis, such methods may still result in false positives due to suboptimal analysis practices and/or the presence of artifacts within the data (Aru et al., 2015; Hyafil, 2015).

### The Limitation of Current PAC Methods

Ideally, the representation of frequency information would not result in confounds of spectral content as a result of factors such as non-sinusoidal waveforms in the signal. However, for most current approaches this does not stand true if the signal contains non-linear waves with non-sinusoidal forms and shapes (for reviews, see Aru et al., 2015; Cole and Voytek, 2017). Standard approaches for computing PAC such as the mean-vector modulation index (Canolty et al., 2006) can be applied across a range of frequency bands in the low frequency phase and high frequency amplitude components to construct a 2-dimensional comodulogram. These measures critically depend on the bandwidth of the filters used in the estimation procedure which may not be obvious from the comodulogram itself. However, they do not involve consideration of the actual nature of the raw signal (i.e., whether it is non-linear and/or non-stationary). Therefore, the theoretical validity of this approach is limited by the fact that it requires an assumption of the signal being both linear and stationary. This ungrounded assumption means such approaches fail to measure non-sinusoidal oscillations, and several studies have demonstrated that spurious PAC values can be seen from non-linear and non-stationary signals *per se* without genuine modulations (Kramer et al., 2008; Penny et al., 2008; Özkurt and Schnitzler, 2011; Kramer and Eden, 2013; Voytek et al., 2013; Pittman-Polletta et al., 2014; Aru et al., 2015; Gerber et al., 2016; Jensen et al., 2016; Lozano-Soldevilla et al., 2016; Cole and Voytek, 2017; Pullon et al., 2019). Although several methods have been proposed to estimate PAC values, none has fully solved these issues (e.g., Canolty et al., 2006; Tort et al., 2010).

### The Aim of the Current Study: Use Holo-Hilbert Spectral Analysis to Fully Measure the Spectral Information of Cross-Frequency Interactions in Neuronal Oscillation Signals

To overcome the limitations of the previous methods, we applied Holo-Hilbert Spectral Analysis (HHSA) as a data-driven method, to investigate complex brain oscillations (Huang et al., 2016; Nguyen et al., 2019; Liang et al., 2021).

Holo-Hilbert spectral analysis is a non-linear analysis tool based on empirical mode decomposition (EMD) to resolve the identification of intrinsic amplitude modulations by representing the data in multiple dimensions (i.e., amplitude modulation, carrier, and time). It should be noted that the carrier frequency of HHSA would correspond to the frequency dimension in conventional spectral analyses. The advantage of EMD is that it can adaptively extract information based on the intrinsic nature of the raw signal (Huang et al., 1998; Sweeney-Reed and Nasuto, 2007) without assumptions of a linear and stationary nature of such a signal. Additionally, the energy content is not restricted by bandwidth selection as it is for current PAC methods. Therefore, this approach is suitable to analyze the spectral properties of non-sinusoidal oscillations and waveform shapes as suggested by Cole and Voytek (2017), van Ede et al. (2018). Based on instantaneous frequency information, HHSA does not merely measure pairwise couplings, but naturally provides energy and content of all possible modulating and carrier frequencies of data resulting from non-stationary and non-linear processes. In addition, the energy of precise frequency values at any time can be extracted to track the temporal characteristics of neuronal oscillations. Therefore, possible types of cross-frequency interactions (inter-mode and intra-mode frequency interaction) and temporal information can be revealed with HHSA.

As mentioned above, for the current PAC analysis methods, a prerequisite for obtaining reliable measures is that the slow- and fast oscillations with their amplitude modulation should appear in the spectral analysis. In the HHSA, these characteristics are presented clearly in a two-dimensional frequency spectrum, in which one dimension is the amplitude-modulating frequency and the other is the frequency of the carrier. For instance, Nguyen and colleagues (Nguyen et al., 2019) used visual stimuli with a 14 Hz carrier and a 2 Hz amplitude modulation to induce steady state visual evoked potentials (SSVEPs) with analysis using Holo Hilbert Spectra. The HHSA outperformed the conventional Fourier approach (i.e., fast-Fourier transform and Bispectrum analysis) by revealing full-dimensional non-linear features and interactions of the induced SSVEPs. This means that HHSA can reveal amplitude modulation occurring in signals recorded from the visual cortex that were induced by entrainment with external visual stimuli (Hyafil et al., 2015). Thus, the current study used HHSA to investigate whether the stimulation by external physical stimuli can dynamically entrain and interact with intrinsic brain waves and generate phase-amplitude couplings.

To examine the variability and reliablity of different analytical methods, we compared the outcomes of FFT analysis, a comodulogram approach and HHSA. These were first applied to a set of controlled simulations of non-sinusoidal waveform shapes. The analysis methods were then applied to SSVEP recordings generated by multiple-input stimulation. The stimulation conditions used in the current study were sinusoidal flicker, amplitude-modulated flicker, and phase-amplitude coupling flicker presenting identical visual stimuli to both eyes (i.e., binocular stimulation). For the human visual system, single-or-multiple frequency input can generate SSVEP responses at the stimulus frequencies and at harmonic frequencies (e.g., Adrian and Matthews, 1934; Norcia et al., 2015; Nguyen et al., 2019). Therefore, our analysis of SSVEP phenomenon with different PAC and HHSA methods aimed to allow assessment of any merits of HHSA for assessment of multiple cross-frequency interactions in comparison to other comodulograms.

## Materials and Methods

### Holo-Hilbert Spectral Analysis

**FIGURE 1:**
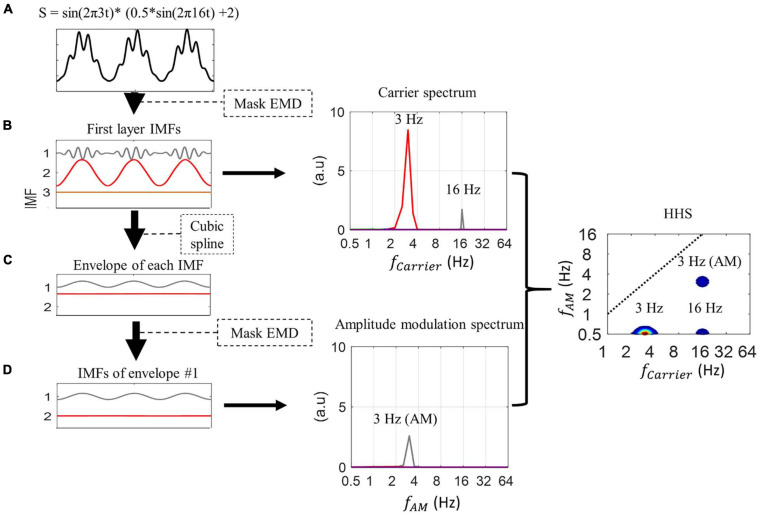
Illustration of the process of Holo-Hilbert spectrum analysis. **(A)** The PAC simulated signal is decomposed into three intrinsic mode functions (IMFs) by Mask EMD to produce the first layer IMFs. **(B)** In this layer, IMF1 corresponds to the high-frequency signal (i.e., amplitude-modulated signal with 16 Hz signal modulated by 3 Hz, see section “Experimental Data”), while IMF2 corresponds to the low-frequency signal (3 Hz). The marginal amplitude spectrum of the first layer IMF shows amplitude peaks at 3 and 16 Hz, respectively. **(C)** The envelope of each IMF is extracted using cubic spline interpolation. The Mask EMD is then applied to each envelope again to acquire second layer IMFs. **(D)** In this case, we only illustrate the second layer IMFs of the first envelope. The amplitude modulation spectrum shows the peak amplitude of IMF1 at 3 Hz, which correspond to the 3 Hz amplitude modulation of the amplitude-modulated input signal. The carrier spectrum and amplitude modulation spectrum are combined to build the two-dimensional frequency spectrum, as known as HHS, in which the *x*-axis represents the carrier frequency (fc), and the *y*-axis represents the amplitude modulation frequency (fam). The HHS shows separate peak amplitudes at 3 Hz (at 0.5 Hz *y*-axis) of the sinusoidal signal, and 16 Hz (at 0.5 Hz *y*-axis) and 3 Hz AM of the amplitude-modulated signal. In the current study, all AM power below 0.5 Hz has been collapsed to the 0.5 Hz AM frequency bin in the HHS. The display of carrier frequencies at 0.5 Hz on the *y*-axis (AM frequency) might not affect the observations of higher AM frequency in the signal. The frequency axes are in dyadic scale.

Holo-Hilbert spectral analysis provides a fully informational spectrum in a two-dimensional frequency representation. That is, both the carrier frequencies (*fc*) and the amplitude modulation frequencies (*fam*) in the signal can be examined simultaneously in the Holo-Hilbert spectrum (HHS) (Huang et al., 2016; Nguyen et al., 2019). To build these axes, two-layer EMD was employed (see the illustration of two-layer EMD in Figure 1). This two-layer EMD was analyzed by a direct quadrature (DQ) transform (Huang et al., 2009) to obtain the instantaneous frequency and amplitude (for details, see Huang et al., 2016; Nguyen et al., 2019; Liang et al., 2021). EMD is a data-driven approach to decompose the signal into several intrinsic mode functions (IMFs) without the selection of band-pass filter cut-offs. Thus, every EMD algorithm serves as a natural dyadic filtering bank (Flandrin et al., 2004), yet it keeps the property of “completeness” and “orthogonality” among IMFs, i.e., the dot product between any two IMFs sufficiently approaching zero and summing over all IMFs reconstructs the original signals. Therefore, in the scheme of instantaneous frequency, the frequency band of each IMF is wide enough to form a continuous band with its previous and next IMFs. This is also why the EMD results could be mapped to a spectral representation. Due to the higher temporal and frequency resolution (achieved by instantaneous frequency defined by the derivative of instantaneous phases) compared to Fourier-based analysis, EMD-based methods (HHT, HHSA) are especially suitable for analyzing non-stationary and non-linear brain signals (Gregoriou et al., 2009; Park et al., 2011; Bajaj and Pachori, 2012; Lopes-dos-Santos et al., 2018). In this study, an enhanced algorithm of EMD, referred to as masking EMD, was used to resolve the mode-mixing problem in the original EMD that might potentially reduce the distortion of HHSA results (Deering and Kaiser, 2005; Tsai et al., 2016; Nguyen et al., 2019). The masking EMD has been proved to be able to robustly decompose the signal into physically meaningful non-linear components (Nguyen et al., 2019). Thus, using masking EMD to implement HHSA also offers a viable method to detect the signals at different noise levels (i.e., SNR = −5, 0, 5, 10) (see the illustration in Supplementary Figure 1).

In general, the analysis flow was conducted as follows:

(1) The original signal *x*(*t*) is decomposed into several IMFs by using masking EMD, with these known as the first layer IMFs, and expressed as follows:

$$x\,\left(t\right)=\sum_{j=1}^{n}c_{\dot{j}}\,\left(t\right)+r_{n}=\sum_{j=1}^{n}a_{\dot{j}}\,\left(t\right)\,\cos\theta_{j}\,\left(t\right)+r_{n}$$

(2) Then the DQ method is applied to estimate instantaneous frequencies and amplitudes of the IMFs. This step gives the time-frequency characteristics of the original signal and is known as the Hilbert-Huang Transform (HHT). To avoid confusion with the terminology regarding frequencies, we will refer to the instantaneous frequency obtained from the first layer EMD as the “carrier frequency” and it is presented along the *x*-axis in the Holo-Hilbert spectrum.
(3) Construct the amplitude function of each given IMF as defined by Huang et al. (2009, 2013, 2013; 2016).
  • Obtain the absolute value of the IMF.
  • Identify all the maxima of the absolute-valued IMF.
  • Assemble the envelope by employing a natural spline through all the maxima.
(4) The second layer EMD is obtained by applying the masking EMD to the amplitude function $a_{\dot{j}}\,\left(t\right)$, given as:

$$a_{j}\,\left(t\right)=\sum_{k=1}^{m}c_{\dot{j\,k}}\,\left(t\right)+R_{j\,m}=\sum_{k=1}^{m}a_{\dot{j\,k}}\,\left(t\right)\,\cos\Theta_{j\,k}\,\left(t\right)+R_{j\,m}$$

⁢ Where $c_{\dot{j\,k}}\,\left(t\right)$ is the second layer IMF, $a_{\dot{j\,k}}\,\left(t\right)$ is the second layer amplitude functions, *cos*Θ_*jk*_(*t*) is the second layer phase function, and *R*_*jm*_ is the trend of each second layer IMF. Thus, the whole expansion of two-layer EMD can be expressed as:

$$x\,\left(t\right)=\sum_{j=1}^{n}\left[\sum_{k=1}^{m}a_{\dot{j\,k}}\,\left(t\right)\,\cos\Theta_{j\,k}\,\left(t\right)+R_{j\,m}\right]\,\cos\theta_{j}\,\left(t\right)+r_{n}$$

(5) The DQ is applied to these IMFs to determine the instantaneous frequency and amplitude of amplitude modulation (*f*_*am*_). The instantaneous frequency and amplitude of this two-layer IMF was projected to (*f*_*am*_, *f*_*c*_, time) space to obtain the three-dimensional Holo-Hilbert Spectrum which describes a complete power spectrum of cross-frequency dynamics varied with time series.
(6) To aid interpretability, the three-dimensional power spectra were marginally summed over the time space to obtain two-dimensional HHS, in which the *y*-axis represents *f*_*am*_, and the *x*-axis shows *f*_*c*_. Supplementary Figure 2 illustrates an example of the frequency resolution of dyadic frequency bands, in which the edges of these bands are constructed by the formula 2^*n*^ (where *n* = −1, 0, 1, 2, 3, 4, 5,…). To assign the power of the carrier and AM frequencies obtained from two-layer IMFs to a specific frequency band (the red rectangle as shown in the Supplementary Figure 2), we marginally summed the power spectra across time points (t) such that 2^5.875^ ≤ *fc*(*t*) ≤ 2^6^ and 2^3.875^ ≤ *fam*(*t*) ≤ 2^4^. Please note that the display of carrier frequencies, which were collapsed across time, at 0.5 Hz on the *y*-axis (envelope frequency) might not affect the observations of higher amplitude modulation in the signal.

### Conventional Phase Amplitude Coupling (PAC) Analysis

In this section, we describe the general steps for measuring the phase-amplitude coupling, using the Modulation Index (MI) value from Tort et al. (2010) as an example. The procedure is outlined in Supplementary Figure 3. The general procedure is separated into four main steps:

(1) The band-pass filter using a Butterworth filter (3rd band-pass filter) is applied to the signal with the region of interest of frequency to extract the slow oscillation (SO) and fast oscillation (FO). The filters for extracting FO need to cover the center frequency ± the SO frequency. We therefore used the variable bandwidth, defined as ±0.333 times the center frequency (Berman et al., 2012; Seymour et al., 2017). In contrast, the bandwidth for SO is set to 1 Hz ± the center frequency.
(2) The Hilbert transform is applied to SO and FO to obtain the instantaneous phase and the amplitude envelope, respectively (Le Van Quyen et al., 2001).
(3) We quantified the coupling measure between SO and FO using the Kullback-Lieber modulation index, as described in Tort et al. (2010). This approach puts the FO amplitude into 18 bins of SO phase. The modulation index is calculated by comparing the amplitude-phase distribution (*P*) against the null hypothesis of a uniformly amplitude-phase distribution (*Q*).
(4) We have performed a block-resampling method to assess the statistical significance of the measured MI values by comparing the raw MI against the distribution of a surrogate dataset, namely surrogated MI (He et al., 2010). That is, the time-series of FO amplitude for each frequency pair was first split into 60 equal-length segments with 50 ms for each segment, and then these segments were shuffled yielding 100 shuffled amplitude time-series in total. This approach preserves the temporal structure of the original signal and therefore is able to produce a rigorous assessments of statistical significance of PAC measures (He et al., 2010; Aru et al., 2015). Finally, the mean and standard deviation of the 100 shuffled MI were computed to obtain a z-score statistic of MI and expressed as:

$$M\,I_{z}=\frac{R\,a\,w\,M\,I-\mu_{s\,h\,u\,f\,f\,l\,e}}{\sigma_{s\,h\,u\,f\,f\,l\,e}}$$

⁢ Due to the expensive computation, we did not use surrogates for the majority of the PAC measures from the synthetic data. To assess the changes of the PAC measures between two conditions across participants, the comodulograms were compared using a distribution of 2000 permutations of non-parametric cluster-based statistics (Maris and Oostenveld, 2007).

### Experimental Data

#### Synthesized Data

##### The General PAC Signal

The general simulated data time-series were generated using the sum of two sinusoidal signals (i.e., SO and FO with its amplitude modulation) (Eq. 1). The phase of SO (f_*P*_) was coupled with the amplitude of FO (f_*A*_) according to Eqs. 2, 3, respectively.

(1) $$x\,\left(t\right)=x_{f\,P}\,\left(t\right)+x_{f\,A}\,\left(t\right)$$

(2) $$x_{f\,P}\,\left(t\right)=A_{f_{P}}\,\sin\left(2\pi f_{P}t\right)$$

(3) $$x_{f_{A}}\,\left(t\right)=A_{f_{A}}\,\left(t\right)\,\sin\left(2\pi f_{A}t\right)$$

Where AfA(t)=Kβsin(2πfA⁢Mt-ϕ)A⁢M.

A_*fP*_ and K are fixed scalars that determine the peak amplitude of SO and FO, respectively. The initial phase of amplitude modulation is ϕ_*AM*_ with a fixed value of −π /2. *f*_*AM*_ indicates the frequency of amplitude modulation. β ∈ [0,1] determines the coupling strength, in which a value of 1 indicates the maximum coupling strength. In all cases of synthesized data, the sampling rate was set to 1000.

##### Non-sinusoidal Simulations

To validate the effect of degree of non-linearity on HHS, we generated a 10 Hz signal with non-linear waveforms. These waveforms were simulated using an analytic formulation of a non-linear wave (Abreu et al., 2010). An oscillatory time-series *X*(t) was generated with:

$$X\,\left(t\right)=U_{w}\,f\,\frac{\left[\sin\left(\omega \,t\right)\,\frac{r\,\sin\phi }{1\,\sqrt{1-r^{2}}}\right]}{1-r\,\cos\left(\omega \,t+\phi \right)}$$

in which *U*_*w*_ represents amplitude, t represents time and ω is frequency. The waveform shape parameters *r*(−1 < *r* < 1) and ϕ $\left(-\frac{\pi }{2}\geq \phi \geq \frac{\pi }{2}\right)$ determine the degree of non-linearity and the direction of skew, respectively. *f* is a function of r controlling amplitude $\sqrt{1-r^{2}}.$ A positive value of ϕ creates an oscillation with a faster rising edge and a slower falling edge and vice versa for a negative value. A value of 0 would yield equivalent rising and falling profiles. The extent of non-sinusoidal features was manipulated by the value of r. and the degree of non-linearity can be parametrically varied to generate a wide range of non-linear waveform shapes (Abreu et al., 2010).

The simulated waveforms with a biologically plausible non-linear waveform with a larger peak than trough and a faster rising edge than falling were generated by fixing ϕ to a value of $\frac{\pi }{4}$. Values of *r* were varied from 0 to 0.9 in steps of 0.1 in which a value of 0 indicates a completely linear (sinusoidal) oscillation and 0.9 is highly distorted. The resulting signals are qualitatively similar to the skewness seen in several types of neuronal oscillation. Thus, the possibility of spurious measures of coupling was emphasized.

#### Electrophysiological Data (Steady-State Visually Evoked Potentials)

##### Participants

Ten students (5 females; mean age = 23.1 years, *SD* = 2.1 years) participated in the first experiment. Eight students (3 females; mean age = 26.1 years, *SD* = 5.4 years) participated in the second experiment. All participants had normal or corrected-to-normal vision and were neurologically healthy. This study was carried out in accordance with the Social and Behavioral Research Ethical Principles and Regulations of National Taiwan University and was approved by the Research Ethics Committee of National Taiwan University. All participants gave written informed consent before participation.

##### Stimuli and Procedures

The stimuli were viewed through two black tubes of 13 cm in length, with one tube for each eye. Each tube contained a white light-emitting diode (LED) covered with a 4 cm × 4 cm diffuser plate at one end of the tube to allow presentation of a stimulus with a visual angle of ∼18.2° and a luminance of up to 39.2 cd/m^2^. The centers of the two tubes were 4.5 cm apart from each other and the device as a whole enabled presentation of different light flicker waveforms for the two experiments in this study. The LEDs were connected to a 16-bit digital-to-analog converter (NI USB-6229 BNC, National Instruments, Austin, TX, United States), allowing the LED signal to be modulated at a rate of up to 40 kHz. An integrated photodiode (BPW34, OSRAM Opto Semiconductors) was used to collect the output LED signal and this was recorded with a BioPac MP35 (Biopac Systems, Inc.) to verify that the emitted signal had the desired shape.

For baseline comparisons, we ran one control condition (no-flicker condition) using a transient flash at the onset and retaining the same luminance across time. We also generated seven testing conditions with sinusoidal flicker, amplitude-modulated flicker and PAC flicker. The frequencies of sinusoidal flicker were set to 3, 5, and 7 Hz. The amplitude-modulated flicker was generated with a 16 Hz carrier and its amplitude modulation, which was of a frequency of 3 or 5 Hz. The PAC flicker was of a frequency of 16 Hz nested in a phase frequency of 3 or 5 Hz. These visual stimuli were generated by using MATLAB (The MathWorks Inc., Natick, MA, United States) in-house programs with the following equations:

$$S\,i\,n\,u\,s\,o\,i\,d\,a\,l\,f\,l\,i\,c\,k\,e\,r:L_{0}+L_{0}\,\left(-\cos\left(2\,\pi \,f_{c}\,t\right)\right)$$

$$A\,M\,f\,l\,i\,c\,k\,e\,r:L_{0}+L_{0}\,\left[\frac{1}{2}\,\left(-\cos\,\left(2\,\pi \,f_{a\,m}\,t\right)+1\right)\,\sin\left(2\,\pi \,f_{c}\,t\right)\right]$$

$$PACflicker:\frac{2}{3}L_{0}+\frac{2}{3}L_{0}[-\cos\left(2\pi f_{a\,m}t\right)\,+\frac{1}{2}\left(-\cos\left(2\pi f_{a\,m}t\right)+1\right)\sin\left(2\pi f_{c}t\right)]$$

Where *t* was a duration of sinusoidal, AM flicker and PAC flicker, L_0_ was the mean of the luminance, *f*_*c*_ was the carrier frequency, and *f*_*am*_ was the modulation frequency.

**FIGURE 2:**
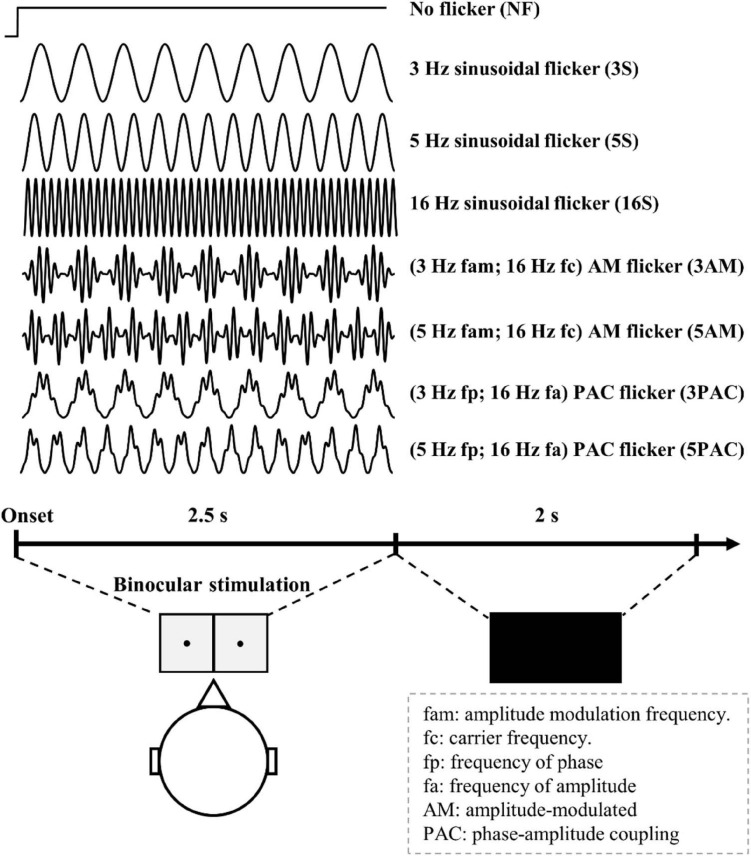
The experimental procedure for Experiment 1. Participants opened their eyes after hearing a beep sound. Various light flicker stimuli were presented to participants in a randomized order with a time duration of 2500 ms for each. After stimulation, participants could close their eyes and rest until the next trial. Light flicker stimulation was presented to both eyes (binocular).

Overall, there was a total 240 trials from eight conditions, in which 30 trials were used for each condition to obtain SSVEP signals (Figure 2). Trials of each condition were randomly presented. Participants were asked to press any key to initiate the first trial. After the keypress, participants were required to open their eyes when they heard a beep sound and then fixate their sight on the black point in the center of the diffuser plate LED for 2.5s. Afterward, participants could take a rest for 2-s (Figure 2). Another beep occurred to indicate the start of the next trial.

#### Electroencephalograms Data Acquisition and Preprocessing

An elastic cap (Electrocap International) containing 36 Ag/AgCl electrodes arranged according to the International 10-20 system was used to obtain the EEG activity which was recorded using a Neuroscan amplifier (Nuamps) and Neuroscan 4.2 software with a sample rate of 1000 Hz. All the data were referenced to the right and left mastoids. The impedance for every electrode was kept below 5 kΩ during the recordings. The continuous data were first filtered with a band-pass filter of 0.5–50 Hz. The data were then epoched from 0 to 3000 ms relative to stimulus onset for each trial and then detrended before excluding trials with blinks or other artifacts (trials with amplitude changes exceeding 100 μV). Afterwards, the preprocessed data were averaged across trials to obtain the SSVEPs. Finally, the SSVEP responses from the Oz channel were used as the main source of the evoked response for further data analysis (Di Russo et al., 2007; Bianciardi et al., 2009; Vialatte et al., 2010). Specifically, we used the SSVEP temporal window from 500 to 2500 ms after the onset of each stimulus to exclude any VEP and to increase the signal to noise ratio of the SSVEP (Andersen et al., 2013; Andersen and Müller, 2015). SPM8 (Wellcome Trust Centre for Neuroimaging^1^), and customized Matlab codes (The MathWorks Inc., Natick, MA, United States) were utilized for further data analysis. The SSVEPs were mainly analyzed with Holo-Hilbert spectral analysis (Huang et al., 2016; Nguyen et al., 2019) to obtain carrier frequencies (*f*_*c*_), and amplitude modulation frequencies (*f*_*am*_). The energy densities are presented by the contour in dyadic frequency scales with eight log2 scale bins (e.g., [8 16] contains eight-frequency bins). In addition, the coupling measure between phase and amplitude oscillations of SSVEPs was also obtained using the Kullback-Lieber modulation index, as described in Tort et al. (2010).

## Results

### Simulations

In Section “Holo-Hilbert Spectral Analysis Can Reflect the Nature of the Non-sinusoidal Signal Without the Presentation of Spurious Coupling as Seen With Conventional Approaches to Measuring PAC,” the simulated non-sinusoidal signals with control of the degree of non-linearity were used to test the effect of the waveform shape on the HHS. In the next two Sections (“The Variations of Coupling Strength Can Be Revealed With HHSA” and “The Time-Variance of Coupling Strength”), the performance of HHSA approach were tested on the simulated PAC signals with fixed coupling strength and time-varying coupling strength. In Section “Multiple Patterns of Cross-Frequency Interaction,” analysis of the synthesized data using HHSA on complex signals with multiple possibilities of cross-frequency interaction was assessed.

#### Holo-Hilbert Spectral Analysis Can Reflect the Nature of the Non-sinusoidal Signal Without the Presentation of Spurious Coupling as Seen With Conventional Approaches to Measuring PAC

Here, the simulated non-sinusoidal signals with specific degrees of non-linearity were used to examine the effects of the waveform shape for different approaches, including HHSA. First, we generated the simulated signals exhibiting non-sinusoidal waveform shape with a frequency of 10 Hz (see Section “Non-Sinusoidal Simulations”).

**FIGURE 3:**
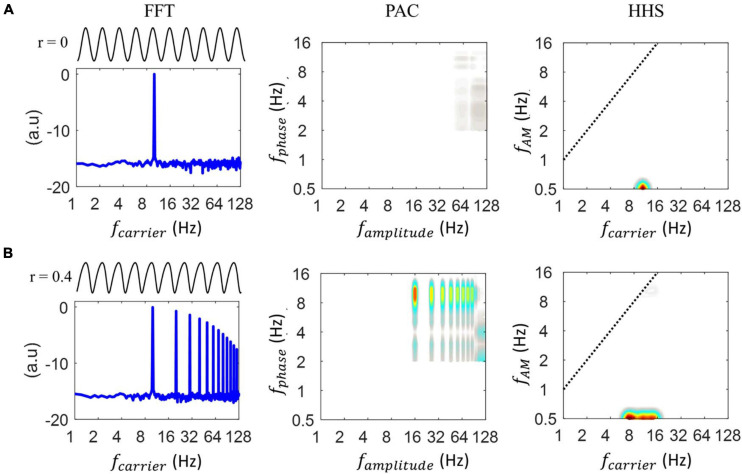
Illustration of how the shape of the waveform alters the resulting phase-to-amplitude comodulogram for different analyses. **(A)** Starting from the left, the first panel shows the 10 Hz sinusoidal oscillation and its FFT. The FFT spectrum shows an amplitude at 10 Hz. Next is shown the PAC comodulogram, estimated by the Modulation Index and finally, the Holo-Hilbert spectrum of the input oscillation is shown. The latter spectrum showed an amplitude increase centered at a 10 Hz carrier without producing harmonics. **(B)** Starting from the left, the first panel shows the 10 Hz non-sinusoidal oscillation, which does not contain any coupling. The FFT spectrum showed an amplitude at 10 Hz and its harmonics. Next, the PAC comodulogram, estimated by Modulation Index, indicated coupling between the 10 Hz phase and 20, 40, and 128 Hz amplitudes, which are all spurious PAC couplings. The Holo-Hilbert spectrum of the input oscillation showed a wider amplitude increase centered at the 10 Hz carrier frequency without any induced harmonics.

Figure 3 displays the outcomes of FFT, PAC and HHSA for the simulated data. When analyzing the sinusoidal signal without the PAC pattern, a clear peak at 10 Hz was present for FFT and HHS (at 0.5 Hz on the *y*-axis) with the absence of a PAC value (Figure 3A). In contrast, when the degree of non-linearity was set to 0.4 (i.e., the signal is now non-sinusoidal), different patterns were seen for FFT, PAC and HHSA. As illustrated in Figure 3B, in addition to the carrier frequency (10 Hz), FFT also displays multiples of the stimulus frequencies, namely the spurious harmonics of the carrier frequency in the spectrum. Critically, the waveform shape, without reflecting the cross-frequency interaction, can exhibit multiple peaks of PAC measures. That is, the 10 Hz phase seems to modulate multiple higher frequency oscillations. The HHSA methods consist of two-layer EMD to establish the two-dimensional frequency spectrum. To further clarify the validation of our proposed approach, we replaced this two-layer EMD with a two-layer band-pass filter and wavelet analysis to obtain the 2D frequency spectrum. Specifically, the band-pass filter and wavelet analysis scans large ranges of carrier (i.e., from 2 to 64 Hz with steps of 2 Hz) and amplitude modulation (i.e., from 2 to 16 Hz with steps of 1 Hz) frequencies. Similar to the results of PAC and FFT, both methods decomposed the non-sinusoidal signals into several higher harmonics in the time-frequency spectrum and the frequency-frequency spectrum as shown in Supplementary Figure 4B. In contrast, the HHSA reflects the non-linear characteristics of the signal with an amplitude increase at a broadband frequency (6-14 Hz, at 0.5 Hz *y*-axis) without any increment of amplitude modulation.

In addition to the above example with degrees of non-linearity, we also discuss another example of non-sinusoidal signal using an exponential non-linearity in the Supplementary Material. That is a 16 Hz FO signal (S) with 3 Hz AM and 0.5 modulation depth occurring as an argument of an exponent of 2 (e.g., 2^*S*^) (Supplementary Figure 5). Thus, the original signal (S) could become the non-sinusoidal signal and consist of the 3Hz SO due to the asymmetry of this signal together with 16 Hz FO and its 3 Hz AM. However, it should be noted that this new non-sinusoidal signal consists of a waveform shape in which the peaks were more narrow/sharper than the troughs. Although the current PAC methods could obtain a true PAC, which was 3 Hz phase coupled with 16 Hz (Supplementary Figure 5B, left and mid panels), this non-sinusoidal waveform shape also produced multiple spurious PAC measures. That is, the 3 Hz phase is coupled with multiple amplitude frequencies of 32, 48, and 64 Hz, etc. (Supplementary Figure 5C, left and mid panels). These results could be also seen in the 2-layer BPF (2L-BPF) and 2-layer WL (2L-WL) analysis (Supplementary Figure 6). In contrast, HHSA can reflect the nature of the non-sinusoidal signal without the presentation of spurious phase-amplitude coupling (Supplementary Figure 5C, right panels). That is, HHSA shows an amplitude increase at 3 Hz SO (at 0.5 Hz *y*-axis) and an amplitude increase at 16 Hz (at 0.5 Hz *y*-axis) with a wideband frequency due to the waveform shape and its 3 Hz AM without the increment of higher frequencies. To further validate the sensitivity of HHSA to noise, we have added the noise levels (i.e., SNR = −5, 0, 5, 10) to this non-sinusoidal PAC signals. HHS was able to detect the coupling at a robust noise (i.e., SNR = −5) while traditional PAC showed no coupling (Supplementary Figure 7). In addition, we also evaluated the effects of noise and data length on the performance of HHSA compared with 2L-BF and 2L-WL (see section “Effect of Data Length and Noise on HHSA” in the Supplementary Material). The results showed that the HHSA, 2L-BF and 2L-WL were affected by noise levels. However, HHSA was less affected by the data length than 2L-BPF and 2L-WL (Supplementary Figure 8).

#### The Variations of Coupling Strength Can Be Revealed With HHSA

To confirm the occurance of phase-amplitude coupling, a spectral peak for the amplitude envelope’s frequency needs to be seen and to match with the frequency of phase (Cohen, 2008; Tort et al., 2010).

**FIGURE 4:**
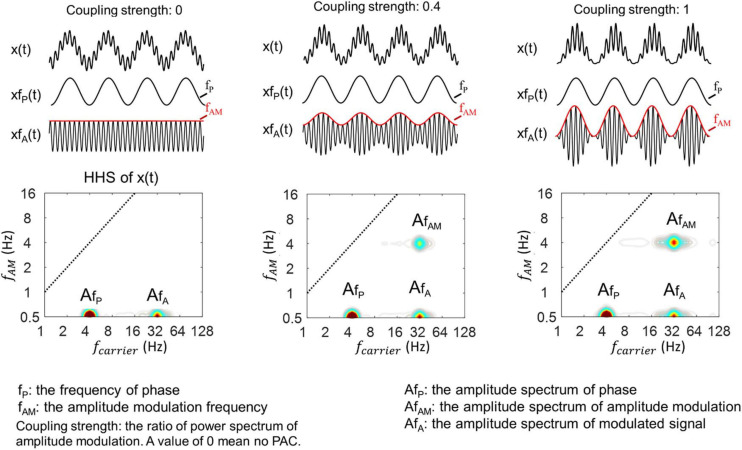
Illustration of HHSA for synthesized data with different coupling strengths, which is the ratio of the modulation depth of xfA(t) in which x(t) = xf_*P*_(t)* xf_*A*_(t). A value of 0 indicates no modulation depth and 1 for full modulation. (Top) 3 levels differing in coupling strength (top trace) along with their corresponding HHS (bottom panels). The modulated signals with fast oscillations are plotted underneath with the amplitude envelope increasing from the top. The synthesized signals all consisted of summation of a 4 Hz sinusoidal signal and an amplitude-modulated signal with 32 Hz modulated by 4 Hz.

The coupling strength, as known as modulation depth in an amplitude-modulated signal, has been suggested to be closely associated with the power spectral density of the amplitude envelope (Tort et al., 2010). Therefore, we tested the sensitivity of the HHSA to track different levels of coupling strength by observing the amplitude envelope’s changes. Here, we mainly used simulated data with theta (4 Hz)-gamma (32 Hz) PAC as examples. Three cases of the synthesized data were generated by controlling the coupling strength with different values of 0, 0.4, and 1 (red line, Figure 4, *top*). A value of 0 indicates no coupling between theta and gamma, and a value of 1 means that the coupling between them is maximal. The HHSA of these data was displayed with the increasing amplitude of amplitude modulation at 4 Hz corresponding for coupling strength of 0, 0.4, and 1 (Figure 4, *bottom*).

In general, the results from HHSA clearly showed the amplitude spectrum of the 4 Hz slow oscillation, 32 Hz fast oscillation and its amplitude modulation at 4 Hz in a two-dimensional frequency spectrum. Moreover, the different levels of coupling strength, as indicated by the amplitude spectrum of AM, are also clearly shown as a result of this analysis.

#### The Time-Variance of Coupling Strength

**FIGURE 5:**
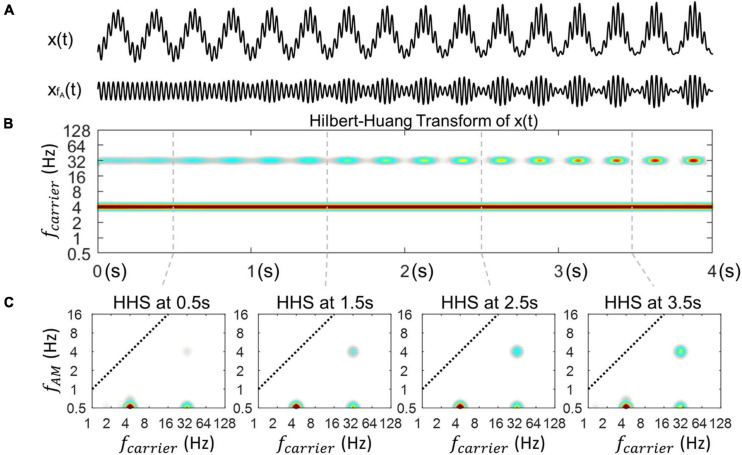
Illustration of the outcome of HHSA on synthesized data with time-varying coupling strength. **(A)** A synthesized signal X(t) with time-varying coupling strength from 0 to 1. The modulated signal (xf_*A*_(t)) shows a power increase corresponding to the coupling strength. **(B)** The time-resolved power spectrum obtained by Hilbert-Huang transform. **(C)** The amplitude spectrum of envelope at 4 time points extracted using Holo-Hilbert spectra to track the 4 various levels of coupling strength across time.

In the previous Section (“The Variations of Coupling Strength Can Be Revealed With HHSA”), the HHSA enabled tracking of the coupling strength on a set of three simulated PAC signals with constant coupling strength over time (Figure 4). In this section, we assess the ability of HHSA to track the time-varying coupling strength where this changed across time in the signal (Figure 5). We used 4s of noiseless synthesized data in coupling strength changed from a value of 0 to 1 over time (Figure 5A). The synthesized data we used contained a 4 Hz phase frequency (f_*P*_) and a 32 Hz amplitude frequency (f_*A*_).

Figure 5B shows an power increase at 32 Hz along with its amplitude modualtion in the outcome of the HHT. In addition, the power of the phase frequency stays unchanged in the HHT spectrum. Next, how the coupling strength changes at specific time points, namely, 0.5, 1.5s, 2.5s, and 3.5s in the HHS is illustrated in Figure 5C. The amplitude spectra of the phase frequency is constant with time whereas the amplitude spectra of the f_*AM*_ frequency are clearly increased at each point, corresponding to the increase of coupling strength.

#### Multiple Patterns of Cross-Frequency Interaction

**FIGURE 6:**
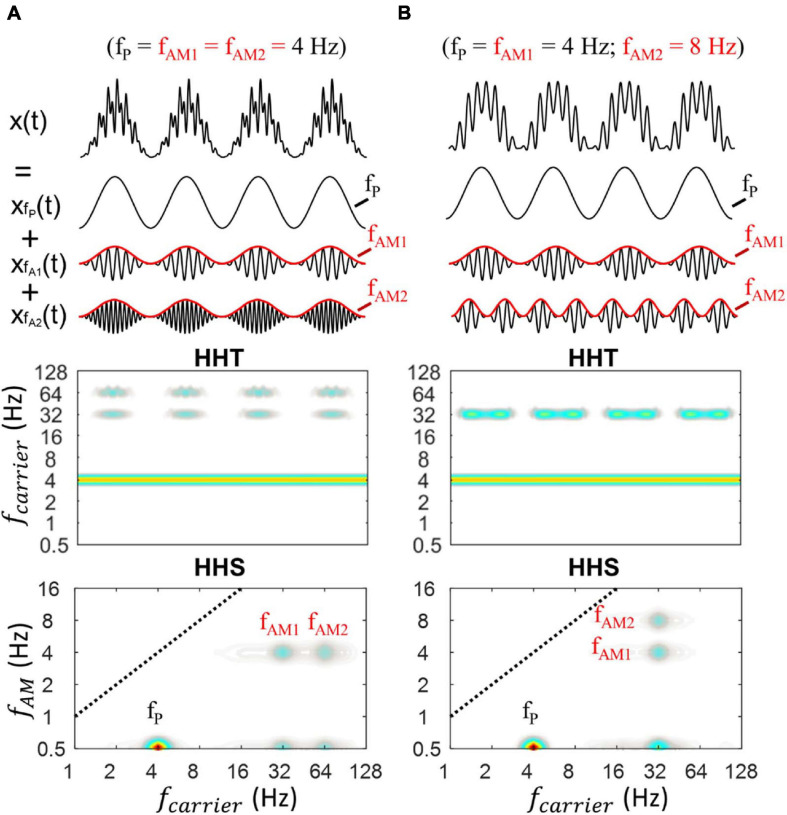
Illustration of HHSA on a synthesized data with the sum of three oscillators (i.e., x(t) = x_*fP(t)*_ + x_*fA1(t)*_ + x_*fA2(t)*_). **(A)** The synthesized PAC data, in which theta phase was coupled with low and high gamma along with their corresponding HHT and HHS (lower panels). The HHT shows time-frequency characteristics of the simulated data, in which the amplitude increases at fP, fA1, and fA2 could be seen over time, corresponding to the original properties of the signals (amplitude modulation). The HHS shows the distinct peaks at f_*P*_, f_*A1*_, f_*A2*_, f_*AM1*_, f_*AM2*_. **(B)** The synthesized PAC data, in which low gamma was modulated by 4 Hz (f_*AM1*_) and 8 Hz (f_*AM2*_) along with their corresponding HHT and HHS (lower panels).

Here, we further evaluate the capability of HHSA in analysis of multiple CFC patterns. We generated two more synthesized data sets made from the sums of three oscillators (i.e., x(t) = x_*fP(t)*_ + x_*fA1(t)*_ + x_*fA2(t)*_). These data allowed two aspects of testing: analysis of (1) low-gamma and high-gamma bands modulated by the same AM frequencies and (2) low-gamma band modulated by the different AM frequencies. In both data, the frequency of phase was set to 4 Hz. Specifically, one noiseless simulated signal was generated with f_*P*_ = f_*AM1*_ = f_*AM2*_ = 4 Hz, f_*A1*_ = 32 Hz, f_*A2*_ = 64 Hz, and the other noiseless simulated signal was generated with f_*P*_ = f_*AM1*_ = 4 Hz, f_*AM2*_ = 8 Hz, f_*A1*_ = f_*A2*_ = 32 Hz. The data length was set to 6s with a sampling rate of 1000 Hz in both cases. The results of HHT and HHSA for both are illustrated in Figure 6.

In Figure 6A, refering to the first case, the HHT shows an amplitude increase at low-gamma (32 Hz) and high-gamma (64 Hz) frequencies with their corresponding amplitude modulation while retaining constant amplitude of theta across time. The HHSA shows simultaneously the amplitude spectra of theta, low gamma, and high gamma at 0.5 Hz on the y–axis. In addition, clear peaks of f_*AM1*_ and f_*AM2*_ can be seen at 4 Hz on the *y*-axis. An extended signal with four peaks of couplings (i.e., 3 Hz phase modulated 16 Hz, 32 Hz, 64 Hz and 128 Hz) was also analyzed with HHSA and traditional PAC. The results showed that with a sinusoidal PAC signal, multiple peaks of couplings could be captured well by both approaches (Supplementary Figure 9). However, when the signal was non-sinusoidal as illustrated in Supplementary Figure 5, it was difficult to distinguish the spurious PACs from the true PAC. In contrast, HHSA can reflect the non-linear characteristics without the presentation of spurious amplitude modulation.

Figure 6B shows another case with only one low-gamma frequency (32 Hz) modulated by the different AM frequencies at 4 Hz and 8 Hz. The HHT shows an amplitude increase at 32 Hz and its corresponding physical meaning while retaining a constant amplitude of theta. The HHS shows the amplitude spectra of theta, low gamma at 0.5 Hz on y–axis. In addition, the distinct peaks of f_*AM1*_ and f_*AM2*_ can also be seen at 4 and 8 Hz on the *y*-axis.

In addition to the above PAC patterns, we also discuss the results of analysis for different AM frequencies (f_*AM*_) with a constant fast oscillation (f_*A*_) in the Supplementary Material. Crucially, the HHSA could track the AM frequencies (f_*AM*_) in these signals (Supplementary Figure 10).

### Steady-State Visual Evoked Potentials Results

Instead of the synthesized data, here real-time EEG data showing the SSVEP phenomenon was analyzed to validate the capability of the HHSA as shown in the simulation data.

#### Experiment 1

##### Physiological Evidence of Phase Amplitude Coupling Is SSVEPs Shown by HHSA

There was a total of seven different conditions in the SSVEP experiments. However, for illustrative purposes, we mainly report four conditions which actually reflect different patterns of amplitude spectra. These are the no flicker condition, 3 Hz sinusoidal flicker (3S), AM flicker with a 16 Hz carrier and its 3 Hz amplitude modulation (3AM), and PAC flicker with 3 Hz phase frequency and 16 Hz amplitude frequency (3PAC). The rest of the conditions (i.e., 5S, 5AM, 5PAC) are shown in Supplementary Figure 11, in which 5S, 5AM and 5PAC also showed amplitude modulation responses similar to 3S, 3AM, and 3PAC, respectively.

**FIGURE 7:**
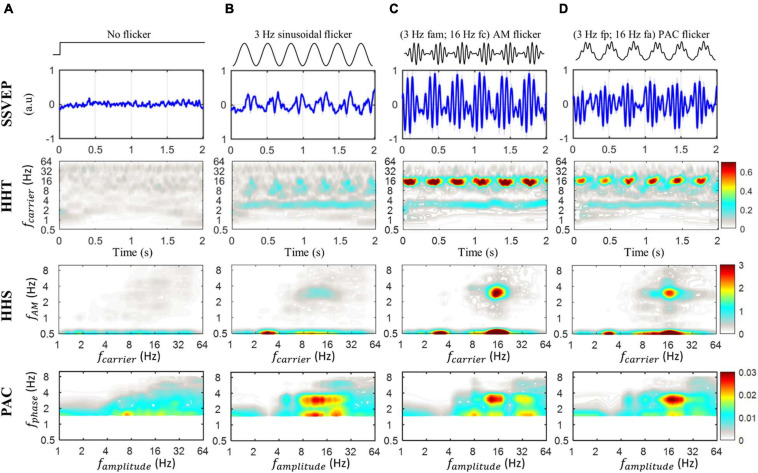
The SSVEP response induced by stimulus with no flicker (baseline), sinusoidal flicker, AM flicker and phase-amplitude coupling flicker, averaged for each condition across subjects for Oz channel recordings. **(A)** The SSVEP response induced by stimulus with no flicker, which was presented at a mean luminance. The amplitude density of the HHT spectrum is unclear. The HHS and comodulogram of the baseline condition (i.e., no flicker condition) also show an unclear pattern of amplitude response. **(B)** The SSVEP response induced by stimulus with 3 Hz sinusoidal flicker. The HHT shows an amplitude increase at the stimulus frequency (i.e. 3Hz) and the higher frequencies (alpha/beta band). In addition, the SSVEP amplitude at higher frequency are modulated by the frequency of the stimulus (3 Hz). In the HHS, the SSVEP amplitudes of stimulus frequency (3 Hz) and 3 Hz AM residing in alpha/beta oscillations are observed. The coupling increase between 3 Hz phase and alpha/beta amplitude can be seen in the comodulogram. **(C)** The SSVEP response induced by amplitude-modulated flicker, which was a 16 Hz carrier and its 3 Hz amplitude modulation. The SSVEP spectrum observed with HHT shows the amplitude increase at 3 Hz and at 16 Hz with its amplitude modulation. The HHS shows the peak amplitudes increase at the 16 Hz carrier frequency (*x*-axis) and its 3 Hz AM (*y*-axis), which correspond to the stimulus frequency. In addition, the peak amplitude at 3 Hz slow oscillation as a non-linear component is also observed. The comodulogram reveals the coupling increase at 3 Hz phase and 16 Hz amplitude. A second coupling increase between 3 Hz phase and 32 Hz amplitude is also observed. **(D)** The SSVEP response induced by phase-amplitude coupling flicker, which was a 16 Hz amplitude frequency nested in a 3 Hz phase. SSVEP spectra observed in HHT show a similar pattern as in panel **(C)**. The HHS shows the peak amplitude increase at 16 Hz carrier (*x*-axis) and its 3 Hz AM (*y*-axis), which correspond to the amplitude-modulated oscillation (or modulated oscillation). In addition, the peak amplitude at 3 Hz oscillation also increases corresponding to the 3 Hz phase oscillation. The 3 Hz phase coupled with 16 Hz can be clearly seen in the comodulogram. A color bar displays z-scores of MI values above the 95th percentile of shuffled distributions (z-score > 1.64).

The HHT as well as PAC and HHSA were first applied to analyze four different sets of data from 10 participants. The results of this analysis are shown in Figure 7. In the no flicker condition, no relevant pattern of amplitude responses was observed from the amplitude density of HHT spectrum, nor in the HHS and PAC (Figure 7A). In the 3 Hz sinusoidal flicker condition (Figure 7B), the amplitude increase at the stimulus frequency (i.e., 3 Hz) and also at higher frequencies (8-16 Hz) were clearly present in the HHT spectrum. In addition, the SSVEP amplitude at higher frequency is obviously seen to be modulated by the frequency of stimulus (3 Hz). Furthermore, the HHS and comodulogram of the SSVEP elicited by 3 Hz sinusoidal flicker are also clearly shown. In the HHS, it is possible to clearly observe the three clear components of the amplitude increase. These are the SSVEP amplitudes at stimulus frequency (3 Hz) and the alpha/beta band (at 0.5 in the *y*-axis) along with 3 Hz amplitude modulation (at 3 Hz in the *y*-axis). Additionally, the coupling increase between the 3 Hz phase and the alpha/beta amplitude can be seen in the comodulogram.

In the case of amplitude-modulated flicker, characterized by 16 Hz carrier and 3 Hz amplitude modulation, the SSVEP responses become more complex than those of 3 Hz sinusoidal flicker (Figure 7C). From the HHT spectrum, we can observe and estimate the SSVEP spectrum with an amplitude increase at 3 Hz and at 16 Hz. In contrast, the HHS shows peak amplitudes increased at the 16 Hz carrier frequency (at 16 Hz in the *x*-axis and 0.5 Hz in the *y*-axis) and its 3 Hz AM (at 16 Hz for the *x*-axis and 3 Hz in the *y*-axis), as well as an amplitude increase in delta frequency (3 Hz in the *x*-axis and 0.5 Hz in the *y*-axis). The comodulogram reveals the coupling increase at 3 Hz phase and 16 Hz amplitude modulation, as well as a second coupling increase between 3 Hz phase and 32 Hz amplitude.

Furthermore, if we generate the SSVEP response to phase-amplitude coupling flicker, which has 16 Hz amplitude nested in 3 Hz phase (Figure 7D) we still observe a similar pattern of SSVEP spectra in HHT as was seen in the case of amplitude-modulated flicker. Crucially, the HHS clearly shows the peak amplitude increase at the 16 Hz carrier frequency (at 16 Hz in the *x*-axis and 0.5 Hz in the *y*-axis) and its 3 Hz AM (16 Hz in the *x*-axis and 3 Hz in the *y*-axis), which exactly correspond to the amplitude-modulated oscillation (or modulated oscillation) of the PAC flicker. The peak amplitude at 3 Hz is also found to increase in the same pattern as the 3 Hz phase oscillation. Further, the delta phase (3 Hz) coupled with beta amplitude (16 Hz) can be clearly seen in the traditional surrogate PAC. In addition, the further analysis using 2L-BPF and 2L-WL also showed results quite similar to those of HHSA (Supplementary Figure 12). However, since PAC, 2L-BPF, and 2L-WL may be affected by the non-sinusoidal signals, these methods were limited in the confirmation of these couplings. In contrast, these couplings were confirmed by the HHSA. Thus, these findings building upon the HHSA method provide clear physiological evidence in support of the existence of phase amplitude coupling in the human brain (or at least in the human visual system).

**FIGURE 8:**
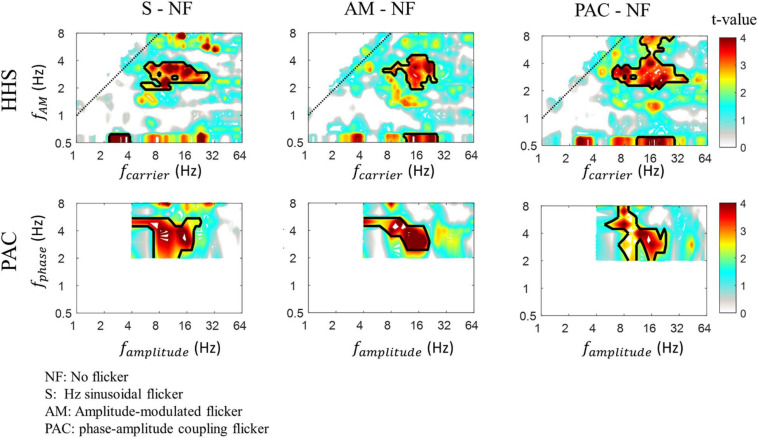
The HHS contrast (top panels) and PAC contrast (bottom panels) of SSVEPs between testing conditions (3 Hz sinusoidal flicker, AM flicker and PAC flicker) and baseline condition (no flicker). The red area within the black contour indicates areas with significant *t*-values (*p* < 0.05, *df* = 9, two-tailed, CBnPP test) of the contrast. The amplitudes of 3 Hz amplitude modulation residing in alpha/beta (8-20 Hz) rhythm are significantly increased.

To confirm the amplitude increase in each flicker condition, we contrasted them to the baseline condition (no flicker condition) using Cluster-based non-parametric permutations (CBnPP). As shown in Figure 8, the CBnPP showed significant increases at 3 Hz amplitude modulation in the alpha/beta band (frequency of amplitude) in three flicker conditions compared to the no flicker condition (*n* = 10, *p* < 0.05, *df* = 9, two-tailed CBnPP). Notably, such a pattern of responses was also defined as the prerequisite for reliably measuring the PAC pattern.

##### Experiment 2: The SSVEPs Elicited by Time-Varying PAC Flicker

**FIGURE 9:**
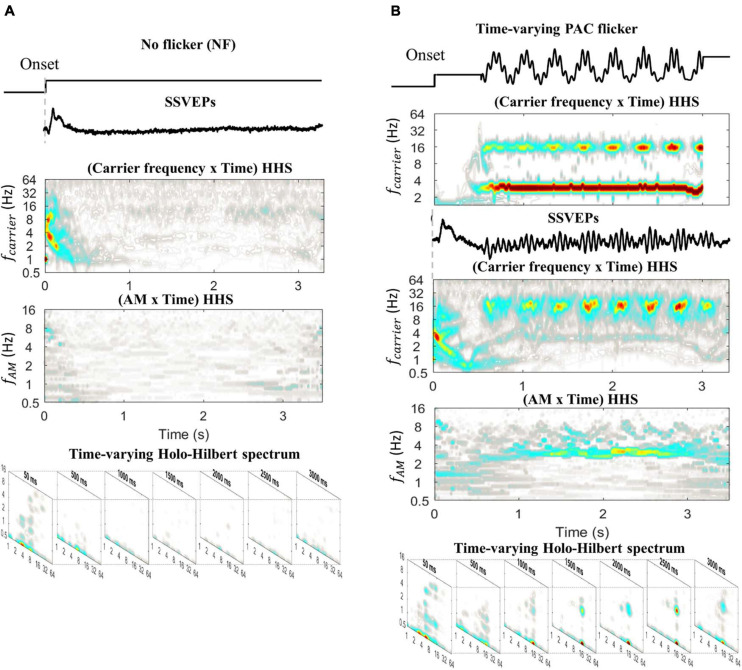
The SSVEPs elicited by no flicker and time-varying PAC flicker, averaged for each condition across subjects. **(A)** The HHT and time-varying HHS analysis of SSVEPs induced by no flicker. The amplitude spectra are unclear for both methods. **(B)** The HHT and time-varying HHS analysis of SSVEPs induced by time-varying PAC flicker. The amplitudes increased at 3 Hz AM modulating 16 Hz carrier corresponding to the stimulation waveform.

In the previous Section (“The Time-Variance of Coupling Strength”), the validation of HHSA was first performed on simulation data. In this section, actual brain data was used to further assess the ability of HHSA to track the time-varying coupling strength (Figure 9). A 3.3 s window (from 0 to 3.3 s relative to the stimulus onset) of SSVEPs for two conditions (50 trials per condition), no flicker (Figure 9A, *top*) and time-varying PAC flicker (Figure 9B, *top*), were included for further analysis. The time-varying PAC flicker contained a constant amplitude of 3 Hz phase-frequency and a time-varying coupling increase of 16 Hz amplitude-frequency. The SSVEPs induced by these conditions were analyzed with HHT and time-varying HHSA. As displayed in Figure 9, the amplitude spectra showed an unclear pattern in the no flicker condition for both methods. In contrast, the amplitudes of the 16 Hz carrier SSVEPs induced by the time-varying PAC flicker condition were better observed by Hilbert-Huang transform and HHSA. In addition, the results using HHSA also showed an amplitude increase over time at 3 Hz amplitude modulation, in which the amplitude was small at the stimulus onset. In sum, the time-varying HHSA successfully captured the dynamic SSVEP response in the actual brain data.

## Discussion

### Summary of Findings

Oscillatory neural dynamics have been commonly considered to be categorized into multiple frequency bands that interact with each other. The current study used Holo-Hilbert Spectral Analysis, which is an EMD-based method, as an alternative to Fourier approaches to explore the cross-frequency interaction of the complex signals. As described in the introduction, the prerequisites to build the real coupling contain at least two features: (1) the frequency of amplitude modulation oscillates at the frequency of phase and (2) the power increase of amplitude modulation. By using HHSA, we found a full dimensional frequency representation of these features from the signals. Although HHSA does not directly measure the pairwise coupling it does provide energy and contents of all possible modulating and modulated frequencies of data resulting from non-stationary and non-linear processes naturally. Thus, HHSA can be beneficial to investigate the cross-frequency interactions of neural oscillations. In this study, we first used simulated data to evaluate the performance of HHSA. The results showed that HHS was able to resolve three main issues: (1) isolation of non-sinusoidal rhythms without harmonic interference, (2) present a high temporal resolution of cross-frequency interaction, and (3) reveal the possible and the concurrent patterns of the cross-frequency interaction. Subsequently, we applied the HHSA to electrophysiological data (SSVEPs). We found an interesting bidirectional coupling phenomenon from the SSVEP responses. These were the SSVEPs in response to 3 Hz sinusoidal flicker driving the alpha/beta oscillation and the SSVEPs induced by AM flicker driving delta oscillation. These findings building upon the HHSA method provide clear physiological evidence in support of the existence of cross frequency interactions. Together, using HHSA, a full spectral representation for the non-linear and non-stationary data can be obtained, with all the possible modes of cross-frequency interaction, both additive and multiplicative, opening a new horizon of analysis of neural processing in the brain.

### Holo-Hilbert Spectral Analysis Shows Meaningful Characteristics of Non-sinusoidal Waveforms Without Harmonics

Since the non-sinusoidal waveform shape, which has the sharpness of peaks or troughs, is an important consideration in phase-amplitude coupling, there is a need for novel methods allowing intuitive exploration of the non-linear and non-sinusoidal features of oscillations as they become prominent in neuroscientific theory (for a review, see Cole and Voytek, 2017). To assess the influence of waveform shape on the results of HHS analysis, the current study employed generated simulated signals with different degrees of non-linearity. Although analysis here was only for some simulations, we expect these results to generalize. The occurrence of spurious PAC means that the power of amplitude modulation residing in fast oscillations is increased and oscillated at the frequency of phase in the absence of fast oscillation. In contrast, HHSA can overcome this limitation, with no PAC pattern introduced when using HHS analysis. The main reason accounting for the spurious values resulting from use of the FFT or PAC method is the linear filter of these methods (Belluscio et al., 2012; Cole et al., 2017) whereas an adaptive filter (i.e., EMD) is used in HHSA, which retains the nature of signal. Finally, the HHSA provides a description of the all amplitude-modulations present within the time-series. Complex patterns of AM might themselves contain multiple frequency components that can be arduous to describe within linear spectra.

### The HHSA Is Able to Capture Possible Cases of Cross-Frequency Interaction

As mentioned by Huang et al. (2016), the HHSA takes advantage of the cross-frequency interactions, in which all possible intra-mode and inter-mode frequency interaction of the complex signal can be presented in a multiple dimensional representation. In the Section “Multiple Patterns of Cross-Frequency Interaction” we generated synthesized data with multiple modes of cross-frequency interaction. The results clearly show that the characteristics of these components can be presented at once in the spectrum. This result demonstrates the capability of HHSA in quantifying multiple modes of cross-frequency interaction. Therefore, it fits the needs of brain investigation to find the signatures of cross-frequency interactions.

For illustrative purposes in the actual brain signal, we also elaborated the detail steps for measuring PAC of the SSVEP induced by 3 Hz sinusoidal flicker using EMD instead of traditional filters (Supplementary Figure 13). We suggest that these steps can be used to obtain the meaningful PAC after detecting the pattern of cross-frequency interaction in HHS results. To evaluate the efficacy of the proposed method, we present clear results from a single participant with a SSVEP with 3 Hz sinusoidal flicker. The HHS results showed a power increase in alpha and beta bands along with their amplitude modulation. Interestingly, the frequency of these amplitude modulations was found to oscillate at 3 Hz (i.e., the stimulus frequency).

In addition, the HHSA was further applied to the SSVEP responses of all participants under four conditions separately: no flicker; 3 Hz sinusoidal flicker; AM flicker; and PAC flicker. The HHS results, averaged across participants, showed that while the SSVEP response to the no-flicker condition had an absence of cross-frequency interaction, the remaining three flickers show patterns of PAC. However, the meaningfulness of these results was different in directional coupling.

In 3 Hz sinusoidal flicker, the amplitude modulation of alpha/beta bands were modulated by the stimulus frequency. That is, this amplitude modulation increased in power and oscillated at stimulus frequency, in this case at 3 Hz. Despite many investigations, SSVEPs at low stimulus frequency (<5 Hz) remain poorly understood. One reason is that the neural activities at these stimulus frequencies have a low signal-to-noise ratio and unexplainable/complicated harmonics (Vialatte et al., 2009). Additionally, the waveform shapes of SSVEPs at low frequencies seem more complex than those of high frequencies which are nearly sinusoidal. In agreement with previous reports, the waveform shape of SSVEPs induced by 3-Hz sinusoidal flicker in the current study was a PAC-like waveform, which was technically observed by HHSA (Figure 7). Since entrainment has been reported as a prominent property of cortical sensory-evoked activity, this PAC-like waveform might be explained by the sensory entrainment theory, in which the slow oscillation drives the fast oscillation (Hyafil et al., 2015). However, the entrainment related to PAC is still under debate since previous studies also suggested that this phenomenon may not reflect actual neural entrainment but might instead be driven by a habituation event-related potential (Mancini et al., 2018; Novembre and Iannetti, 2018). In an attempt to resolve this issue, we analyzed the HHSA of the first VEPs of the no flicker and 3 Hz sinusoidal flicker conditions (from 0 to 300 ms related to the stimulus onset). The results showed distinctive HHSA patterns between transient VEPs and SSVEPs (Supplementary Figure 14), indicating the entrainment related to PAC might not be a consequence of VEPs in the 3 Hz sinusoidal flicker condition. That is, the HHS of SSVEPs showed amplitude increases at the 3 Hz amplitude modulation residing in alpha/beta frequencies and the 3 Hz fundamental frequency (i.e., carrier frequency) indicating the coupling patterns. In contrast, the HHS of VEPs showed an unclear amplitude modulation. In addition, the VEPs in both conditions showed a similar amplitude increase in the time domain and the time-frequency domain, indicating the same pattern of neural oscillations at stimulus onset. While the theoretical work focuses on the coupling of individual neurons, the current results add one more valuable piece of evidence indicating sensory entrainment related to PAC reflected by EEG in the human visual cortex, in which the fast oscillation was modulated by the slow “external sensory” oscillation. From the current results we speculate that the sensory entrainment approach may pave the way to pinpoint more specific CFC patterns by manipulating the waveform shape of the visual stimuli.

Besides the neural entrainment related to PAC of slow external sensory oscillations, we found the opposite directional coupling for the fast external sensory oscillation, which is AM flicker characterized by a 16 Hz carrier and its amplitude modulated at 3 Hz, drives the slow internal oscillation (3 Hz). A similar direction, with the fast frequency driving the low frequency, has been also reported Jiang et al. (2015). They claimed that the envelope of gamma oscillations could drive the alpha phase. Together, the current findings imply that directional coupling can be considered as a potential index to understand the mechanism of neural oscillations and the results obtained demonstrate that the steady-state PAC can be revealed and detected by HHSA.

### The Importance of Time-Varying HHS Analysis

The neural oscillations of the brain are complex and are usually recorded with a high-temporal resolution, typically in the millisecond range. As the pattern of phase-amplitude coupling may vary frequently over time or only be present at specific intervals, temporal resolution is an important factor to characterize the dynamics of coupling. The current PAC methods enable us to apply time-windowed analysis to show temporal dynamics in the coupling. However, since both the phase and amplitude measures are obtained by convolutional integrals using a bandpass filter, the temporal resolution may be low or require a reasonable time window to capture the precise onset of task. In contrast, the results in the current study suggest that HHS analysis can be used to successfully track the time-varying coupling strength at each time point of the signal. While traditional methods calculating cross-frequency interactions have poor temporal resolutions, recent studies have attempted to fill this gap by calculating the short-time PAC or instantaneous PAC (Samiee and Baillet, 2017; Martinez-cancino et al., 2018). Even though the performance of these methods presents an advantage in quantifying the high-temporal PAC value in sinusoidal simulations, whether this is the case for non-sinusoidal simulations or waveform shapes remains to be proven. Since the linear filter used in these methods may lead to spurious or uncertain PAC, it is still necessary to carry out further tests for such signals. As an alternative method to extract the characteristics of instantaneous cross-frequency interaction, HHS analysis is expected to allow tracking of dynamic cross-frequency interactions over time.

## Conclusion

The present study demonstrates the capability of HHSA in extracting possible cases of cross-frequency interaction with a high-temporal resolution. Moreover, this novel method is also suitable for exploration of the non-linear and non-sinusoidal features of oscillations which have become prominent in neuroscientific theory. Interestingly, in collected physiological data, the bidirectional coupling between delta and alpha/beta band can be seen using HHSA, confirming physiological evidence of cross-frequency interactions in the human brain. These findings not only validate the efficacy of the HHSA in revealing the natural characteristics of signals, but also shed more light on further applications in analysis of human brain electrophysiological data with the aim of understanding the functional role of neuronal oscillations in different cognitive functions.

## Data Availability Statement

The raw data supporting the conclusions of this article will be made available by the authors, without undue reservation.

## Ethics Statement

The studies involving human participants were reviewed and approved by the Research Ethics Committee of National Taiwan University. The patients/participants provided their written informed consent to participate in this study.

## Author Contributions

C-HJ, KN, NM, and W-KL designed the experiments. KN and Y-HC collected the data. C-HJ, KN, W-KL, AQ, Y-HC, J-RY, and NH analyzed the data. All authors wrote the manuscript.

## Conflict of Interest

The authors declare that the research was conducted in the absence of any commercial or financial relationships that could be construed as a potential conflict of interest.

## Publisher’s Note

All claims expressed in this article are solely those of the authors and do not necessarily represent those of their affiliated organizations, or those of the publisher, the editors and the reviewers. Any product that may be evaluated in this article, or claim that may be made by its manufacturer, is not guaranteed or endorsed by the publisher.

## Footnotes

1
https://www.fil.ion.ucl.ac.uk/spm/
