# Supplementary material for: Revealing the Dynamic Nature of Amplitude Modulated Neural Entrainment With Holo-Hilbert Spectral Analysis
Source: Front Neurosci. 2021 Aug 5;15:673369. doi: 10.3389/fnins.2021.673369 (PMC8375503; doi:10.3389/fnins.2021.673369)
Supplement: Supplementary file 1 [file Data_Sheet_1.pdf]

Supplementary Material

1. Supplementary Results

1.1. Frequency-varying amplitude modulation (AM)

To assess the ability of HHS to track the possible cases of cross-frequency interaction in complex oscillations we analyzed 6s lengths of data with three different cases of AM frequencies (f_{AM}), with frequencies at 1 Hz, 4 Hz and 7 Hz (**Figure S10**) while the frequency of phase (fP) and the frequency of amplitude (fA) were consistently retained at 4 Hz and 64 Hz, respectively.

Figure S10A shows the HHT and HHS analysis on the first case of a complex oscillation for f_P = 4 Hz and f_{AM} = 1 Hz. The HHT spectrum clearly shows the amplitude change of the x_{fA} signal at 64 Hz corresponding to its physical meaning while retaining the amplitude spectrum of theta phase at 4 Hz over time. In HHS, the amplitude spectra at f_P, f_A and f_{AM} frequencies were simultaneously observed.

In a similar manner, when the f_{AM} frequencies were set to 4 Hz (**Figure S10B**) and 7 Hz (**Figure S10C**), the HHT analysis also showed the amplitude changes at the high frequencies. Crucially, the HHS could track the AM frequencies (f_{AM}) in these signals.

1.2. Using Empirical Mode Decomposition to measure Modulation Index (MI)

We used the IMFs instead of a band-pass filter to calculate the MI measure of the trial-mean SSVEP elicited by 3 Hz sinusoidal flicker from a single subject. **Figure S13** illustrates the steps to calculate the MI value. We used 3 Hz sinusoidal flicker to induce the SSVEPs for illustration purposes. (**Figure S13A**, *left panel*). HHS results for the photodiode signal show only one peak at 3 Hz (on the 0.5 Hz y-axis) (**Figure S13A**, *middle panel*). The PAC method using Tort et al 2010 's method was also used to verify this pattern (**Figure S13A,** *right panel*). The comodulogram of MI showed unclear pattern since the visual stimulus only contains the frequency of phase (i.e., 3 Hz sinusoidal flicker). The SSVEPs elicited by 3 Hz sinusoidal flicker and its Fourier spectrum are shown in **Figure S13B** (*left panel*). The HHS result shows the two peaks of amplitude modulation at 3 Hz (y-axis) in the alpha and beta bands (**Figure S13B**, *middle panel*). The frequency of amplitude modulation is similar to the frequency of phase (stimulus frequency at 3 Hz). This result satisfied the prerequisites of coupling occurrence. However, the comodulogram showed only one peak of PAC value, in which the delta phase (3 Hz) is coupled in the beta band (**Figure S13B**, *right panel*). To evaluate this observed coupling, the SSVEP was first decomposed into several IMFs using EMD**.** **Figure S13C** displays three IMFs (i.e., IMF4, IMF3 and IMF2), in which each IMF contains each frequency band. The amplitude envelopes of these IMFs were also extracted using cubic interpolation. In addition, the instantaneous phase of the delta-IMF (IMF5, peak at 3 Hz), which was extracted by Hilbert transform, was coupling with the amplitudes of high-frequency IMFs (i.e., IMF4, IMF3, and IMF2).

1.3. Effect of Data Length and noise on HHSA

To investigate the validity of the three approaches (i.e., HHSA, 2L-BPF and 2L-WL), we mainly used the amplitude-modulated (AM) signals (i.e., 3 Hz modulate 16 Hz) with added noise and controlled signal length. That is, signal-to-noise ratio (dB) was controlled in the range from -9 to noiseless and the signal length was an integer multiple (from 2 to 10) of AM cycles (3Hz). In this case, as shown in **Figure S8**, we found that the 3 Hz AM power of these were affected by noise levels. However, the 3 Hz AM power of HHSA was less affected by the trial length than for bandpass filter and Wavelet. For example, at the short length of data (2 x 3 Hz cycle length), the 3 Hz AM powers obtained by the bandpass filter and wavelet analysis were smaller than those at the longer data length

(> 2 x 3 Hz cycle length) while the 3 Hz AM powers obtained by HHSA were retained consistently across data length.

2. Supplementary Figures

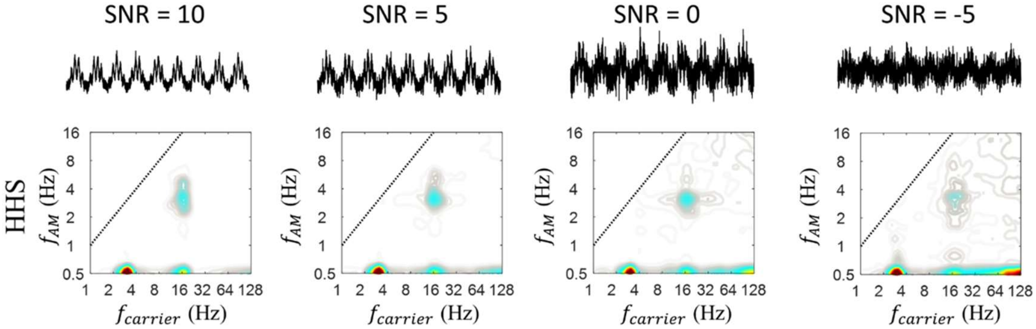

Figure S1. The HHS of sinusoidal PAC signals at different noise levels (i.e., SNR = -5, 0, 5, 10).

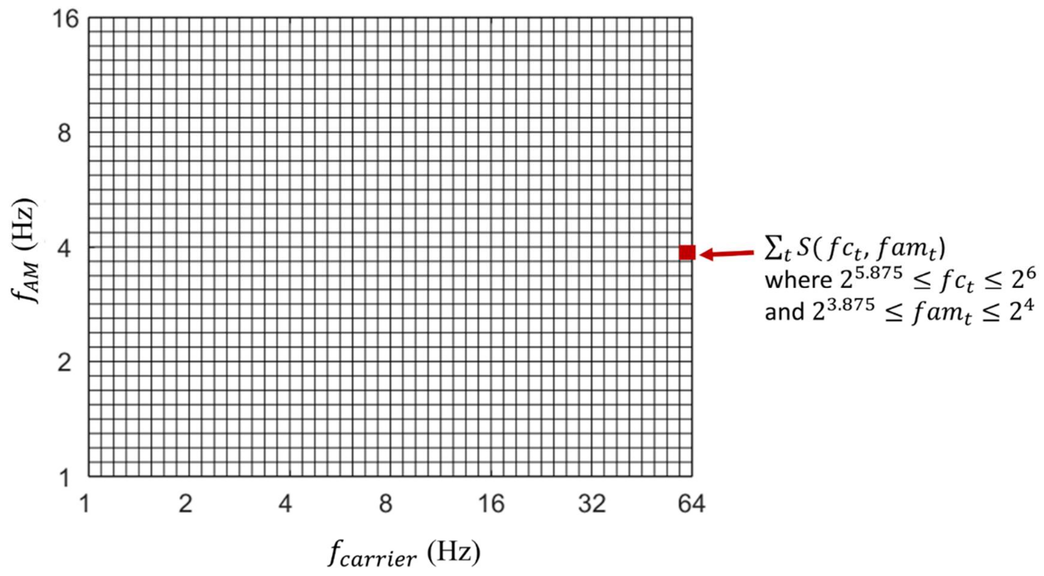

Figure S2. The illustration of dyadic resolution for HHSA. Each dyadic frequency band consists of 8 sub-frequency bins.

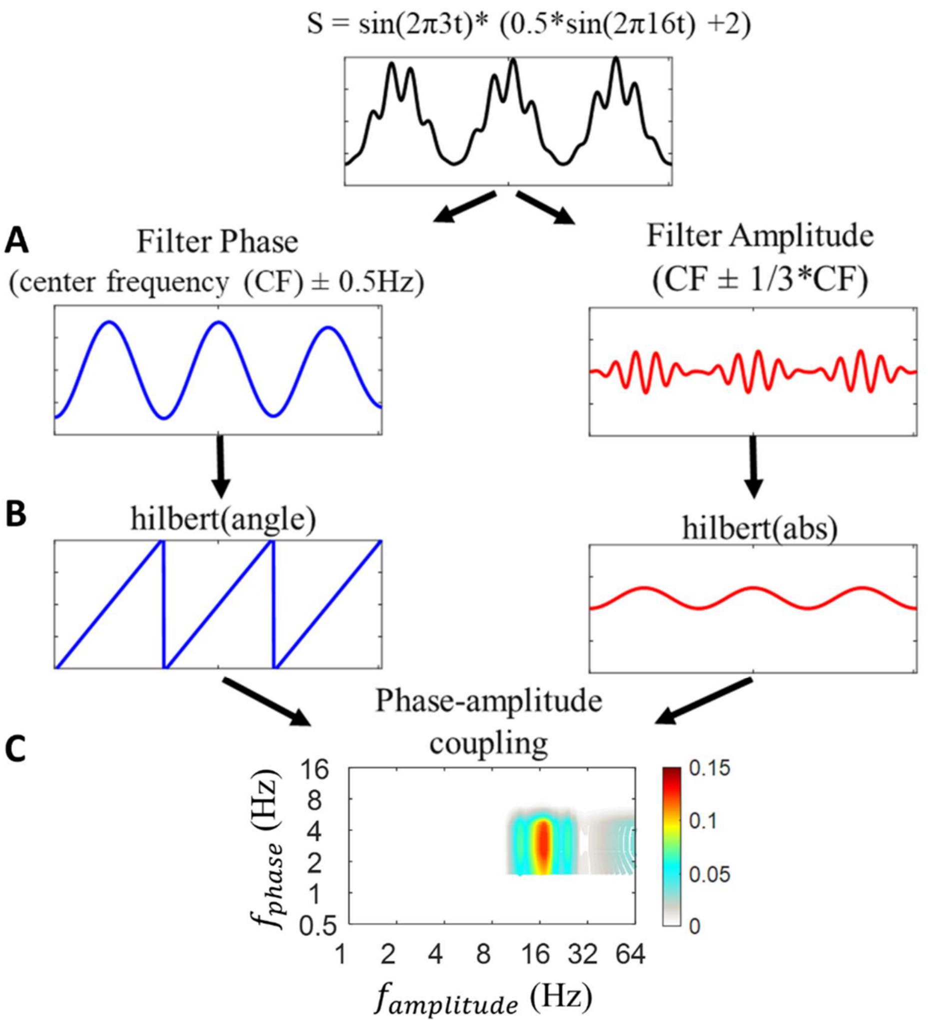

Figure S3. **Procedure for calculating the phase-amplitude coupling.**

A simulated signal was generated by adding the sinusoidal signal of 3 Hz with the amplitude-modulated signal, which was composed of 3 Hz modulating a 16 Hz fast oscillation.

A. The slow oscillation and fast oscillations are extracted by the 3rd Butterworth band-pass filter with the pre-defined frequency band (i.e., center frequency (CF) ± 1 for slow oscillations and CF ± CF/3 for fast oscillations).

B. The phase of slow oscillations and envelope amplitude of fast oscillations are obtained using Hilbert transform.

C. To measure the coupling strength between phase and amplitude oscillations, the modulation index value (MI) is used and quantified using the Mean Vector Length (Tort et al., 2010). The x-axis is the frequency of amplitude and the y-axis is the frequency of phase.

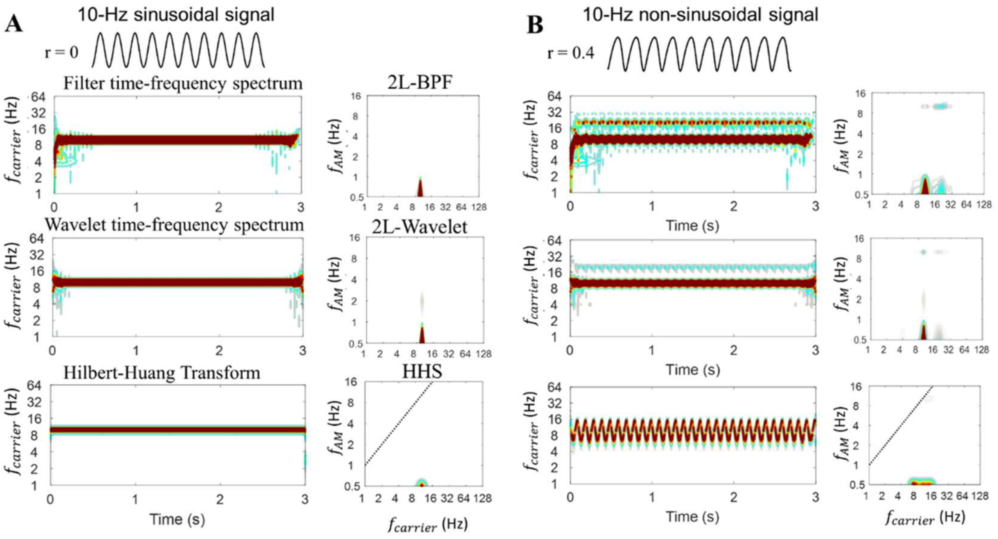

Figure S4. **Illustration of the shape of the waveform for different analyses.**

A. Starting from the left, the first panel shows the 10 Hz sinusoidal oscillation (r = 0) and its power spectra in the time-frequency domain and the frequency-frequency domain.

B. Starting from the left, the first panel shows the 10 Hz non-sinusoidal oscillation (r = 0.4), which does not contain any coupling. The 2L-BPF and 2L-Wavelet spectra showed an amplitude at 10 Hz and its harmonics. The Holo-Hilbert spectrum of the input oscillation showed a wider amplitude increase centered at the 10 Hz carrier frequency without any induced harmonics.

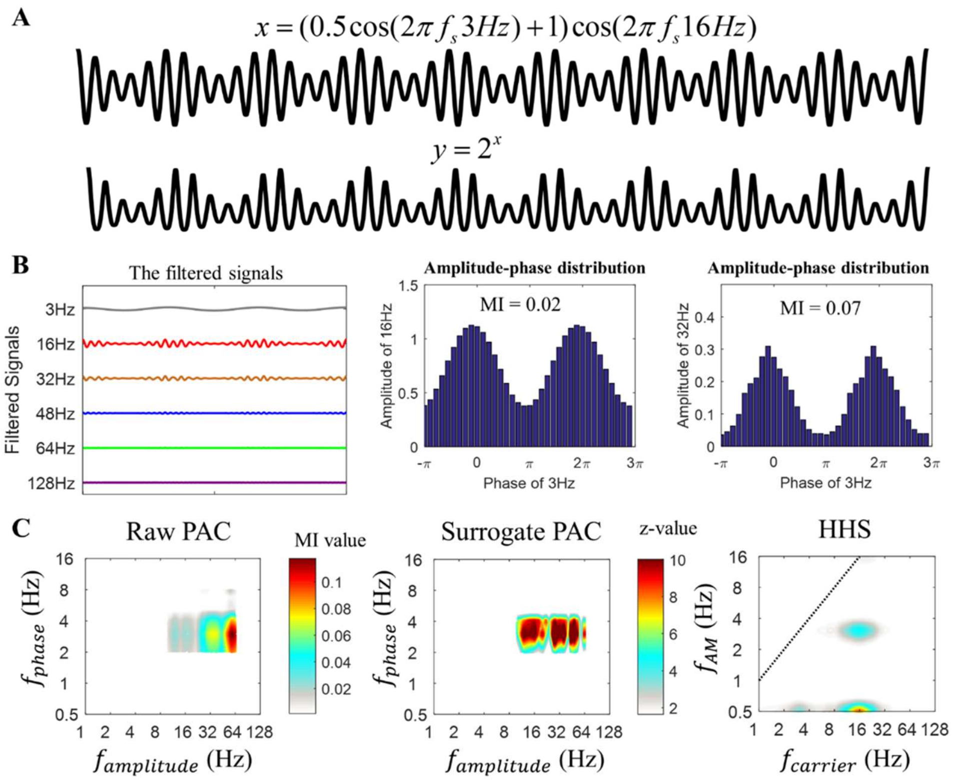

Figure S5. Illustration of a non-sinusoidal signal using exponential nonlinearity.

A. The synthesized signals all consisted of a 16 Hz FO signal with 3 Hz AM and 0.5 modulation depth (S) occurs as an argument of an exponent of 2.

B. Starting from the left, the first panel shows the filtered signals at 3 Hz SO and higher amplitude frequencies. Next, is shown the amplitude-phase distribution of 3 Hz phase and 16 Hz amplitude. The latter amplitude-phase distribution of 3 Hz phase and 32 Hz amplitude showed a spurious MI value of 0.07.

C. Starting from the left, the first and second panels show the raw and surrogate PAC comodulograms, respectively. The latter spectrum of HHSA showed wider amplitude increases centered at a 16 Hz carrier and its 3Hz AM without producing harmonics.

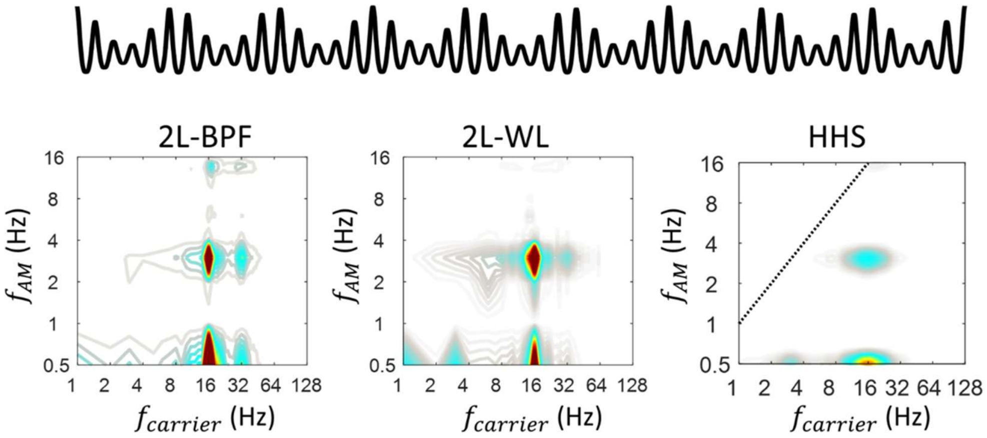

Figure S6. Illustration of a non-sinusoidal signal using exponential nonlinearity. In comparison with HHS, 2L-BPF and 2L-WL showed spurious and diffused couplings in the results.

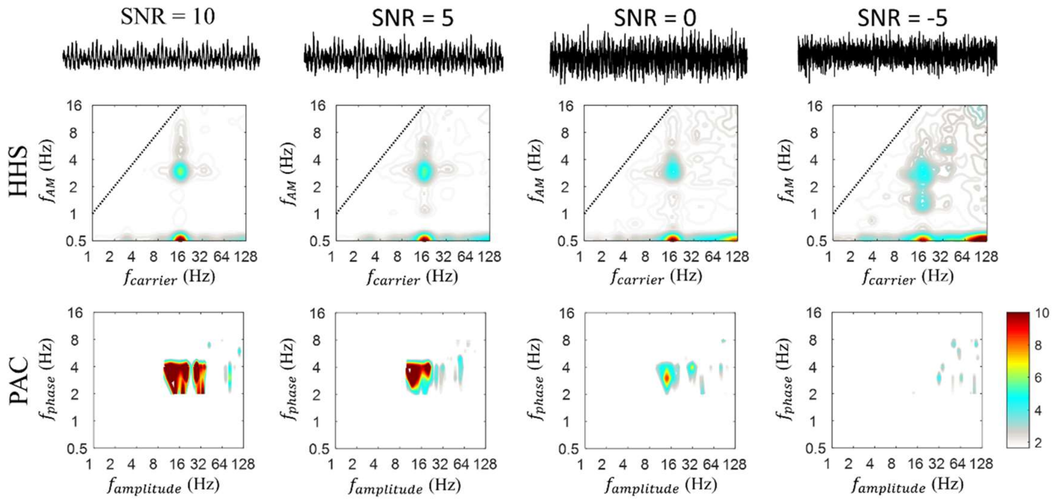

Figure S7. The HHS and comodulograms of non-sinusoidal PAC signal at different SNR levels.

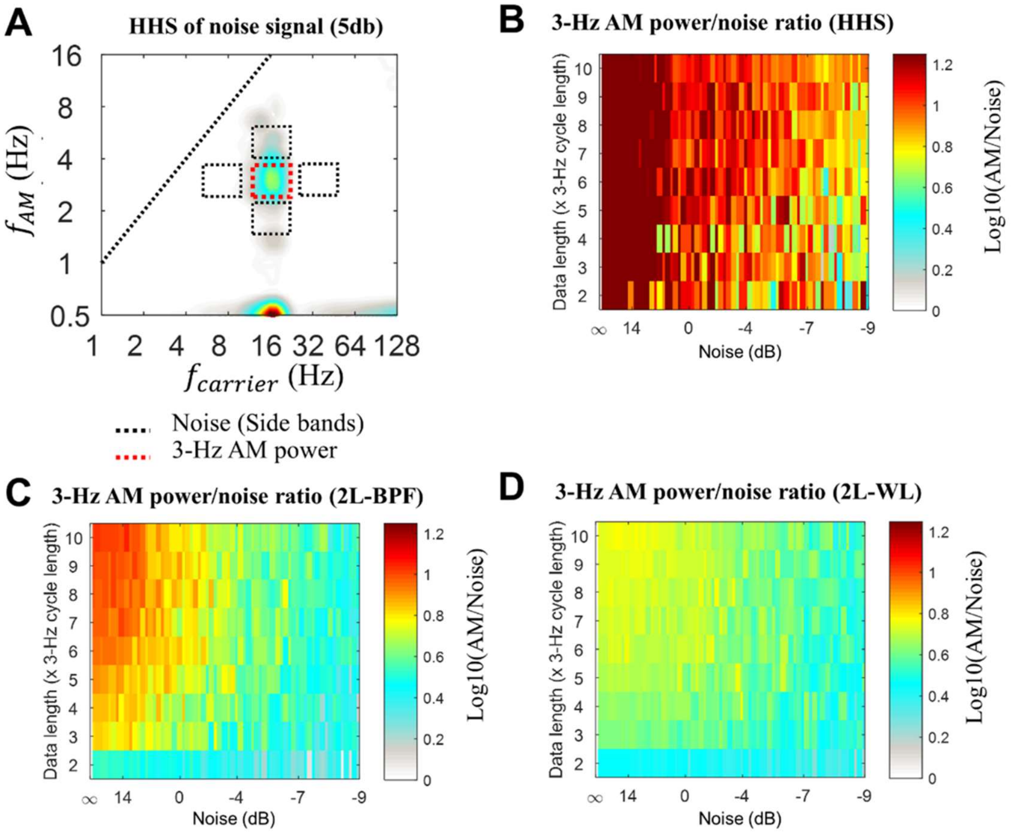

Figure S8. The evaluation of different approaches (HHSA, 2L-BPF, 2L-WL) for different noise levels (resulting in different SNRs), ranging from 8 dB to -9 dB, and data lengths. A. The HHS of noise signal (5 dB). B. The signal-to-noise ratio of 3 Hz AM power measured by the ratio of 3 Hz AM power (red rectangular dashed line) to mean noise power (black rectangular dashed line), obtained by HHSA. C and D show the signal-to-noise ratio of 3 Hz AM power obtained by 2L-BPF and 2L-WL, respectively.

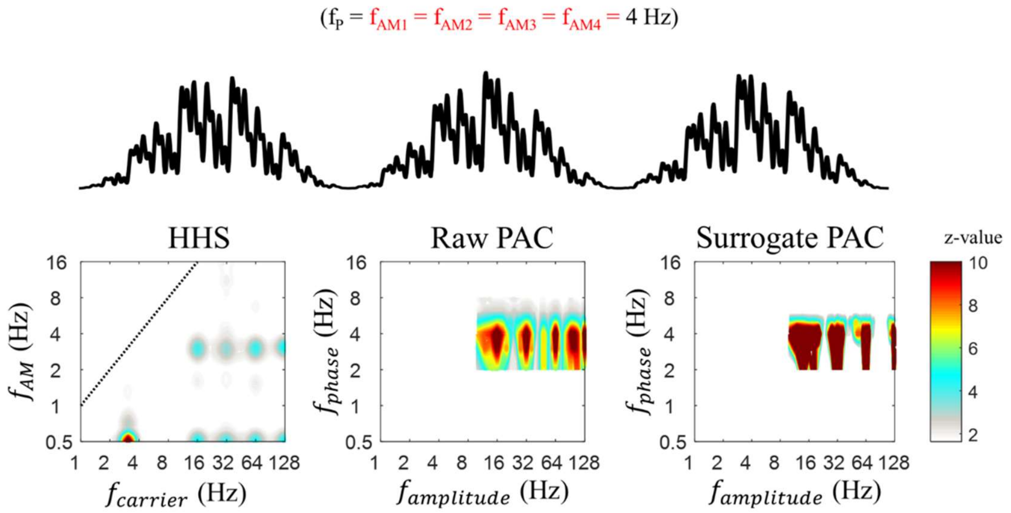

Figure S9. Illustration of HHSA and traditional PAC on a synthesized data with the sum of five oscillators (i.e., $x(t) = x_{fP(t)} + x_{fA1(t)} + x_{fA2(t)} + x_{fA3(t)} + x_{fA4(t)}$). The synthesized CFC data, in which 3Hz phase was coupled with amplitudes of 16 Hz, 32 Hz, 64 Hz and 128 Hz along with their corresponding HHS (bottom left panel) and PAC (bottom mid and right panels). The HHS and PAC methods show the distinct peaks at f_P, f_{A1}, f_{A2} f_{A3}, f_{A4}, f_{AM1}, f_{AM2}, f_{AM3}, f_{AM4}.

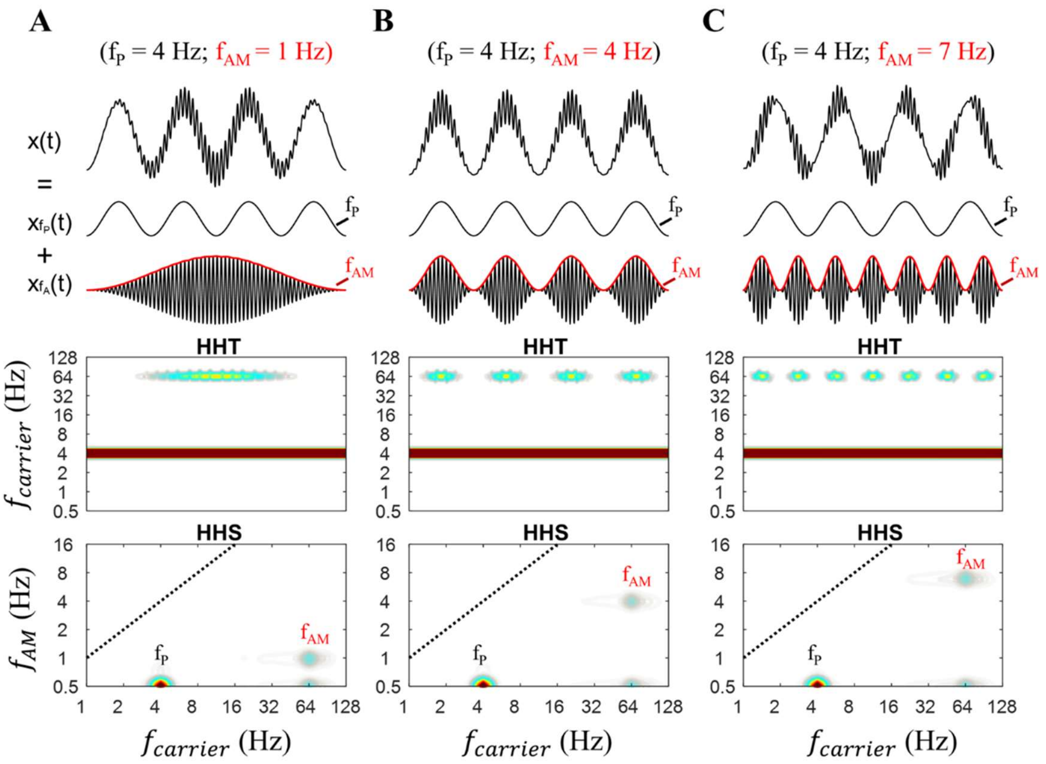

Figure S10. Illustration of HHSA results on synthesized data with the three modulating frequencies (f_{AM}) of high-frequency oscillations.

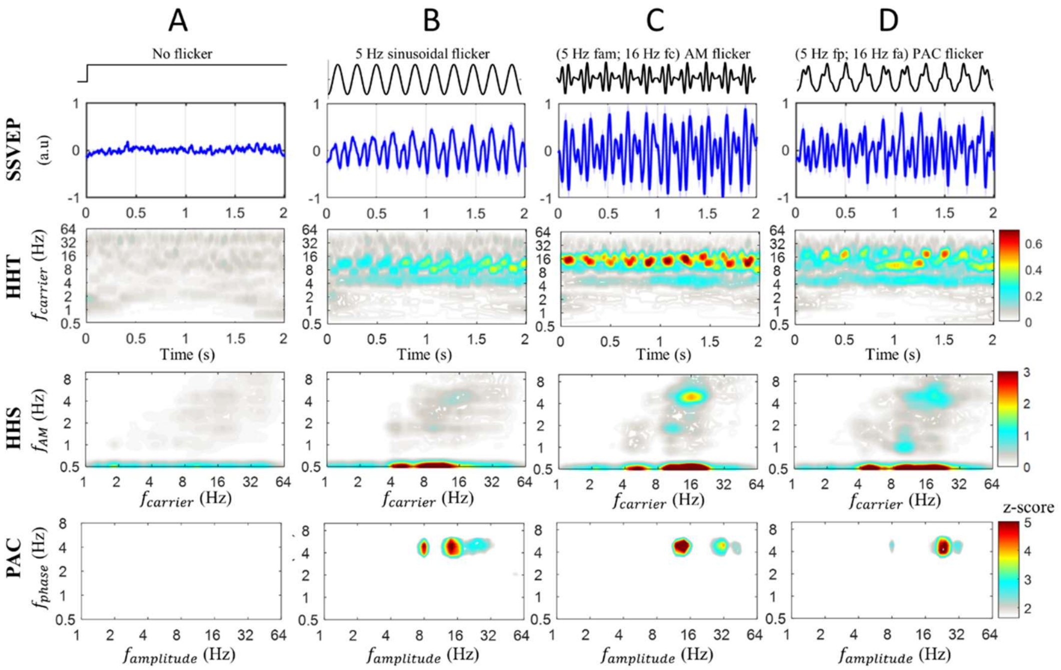

Figure S11. The SSVEP response induced by stimulus with no flicker (baseline), 5-Hz sinusoidal flicker, (5 Hz:16 Hz) AM flicker and 16-Hz amplitude nested in a 5-Hz phase flicker, averaged for each condition across subjects for Oz channel recording.

A. The SSVEP response induced by stimulus with no flicker.

B. The SSVEP response induced by stimulus with 5 Hz sinusoidal flicker.

C. The SSVEP response induced by amplitude-modulated flicker, which was a 16 Hz carrier and its 5 Hz amplitude modulation.

D. The SSVEP response induced by phase-amplitude coupling flicker, which was a 16 Hz amplitude frequency nested in a 5 Hz phase. A color bar displays z-scores of MI values above the 95th percentile of shuffled distributions (z-score > 1.64).

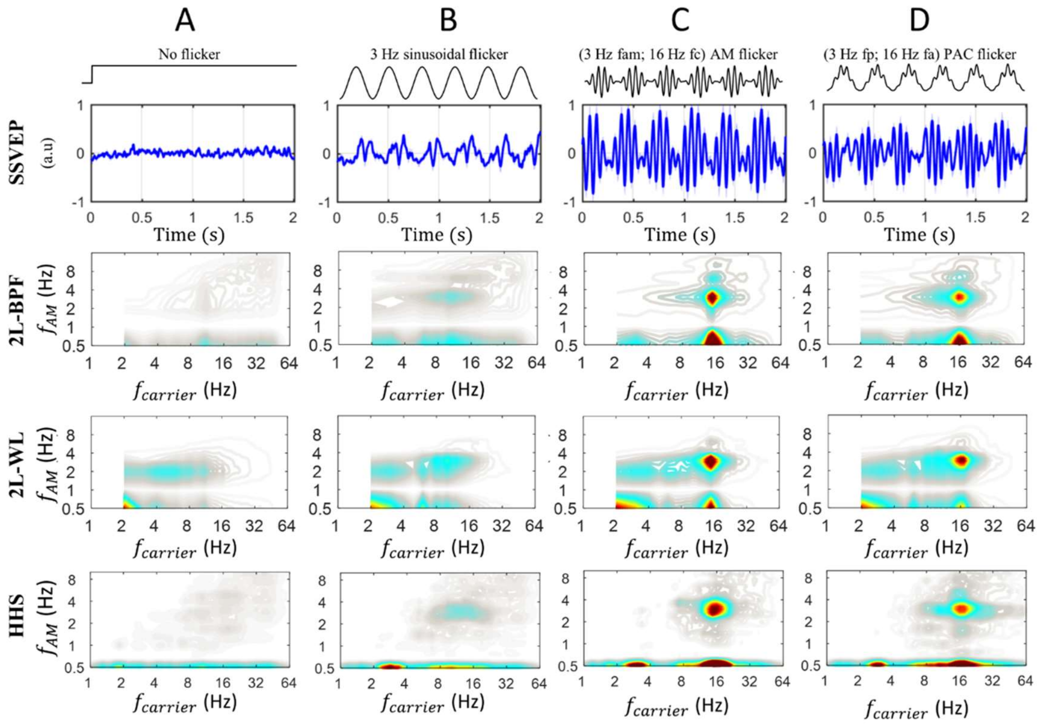

Figure S12. The SSVEP response induced by stimulus with no flicker (baseline), sinusoidal flicker, AM flicker and phase-amplitude coupling flicker, averaged for each condition across subjects for Oz channel recordings.

A. The SSVEP response induced by a stimulus with no flicker. The HHS, 2L-BPF, 2L-Wavelet of the baseline condition (i.e., no flicker condition) also show a diffused pattern of amplitude response.

B. The SSVEP response induced by a stimulus with a 3 Hz sinusoidal flicker. The SSVEP amplitudes of the stimulus frequency (3 Hz) and 3 Hz AM residing in alpha/beta oscillations are observed in the three approaches. The coupling increase between 3 Hz phase and alpha/beta amplitude can be seen in the comodulogram.

C. The SSVEP response induced by amplitude-modulated flicker, which was a 16 Hz carrier and its 3 Hz amplitude modulation. The peak amplitude of 3 Hz slow oscillation as a non-linear component is also observed.

D. The SSVEP response induced by phase-amplitude coupling flicker, which was a 16 Hz amplitude frequency nested in a 3 Hz phase. The peak amplitude at 3 Hz also increases corresponding to the 3 Hz phase oscillation.

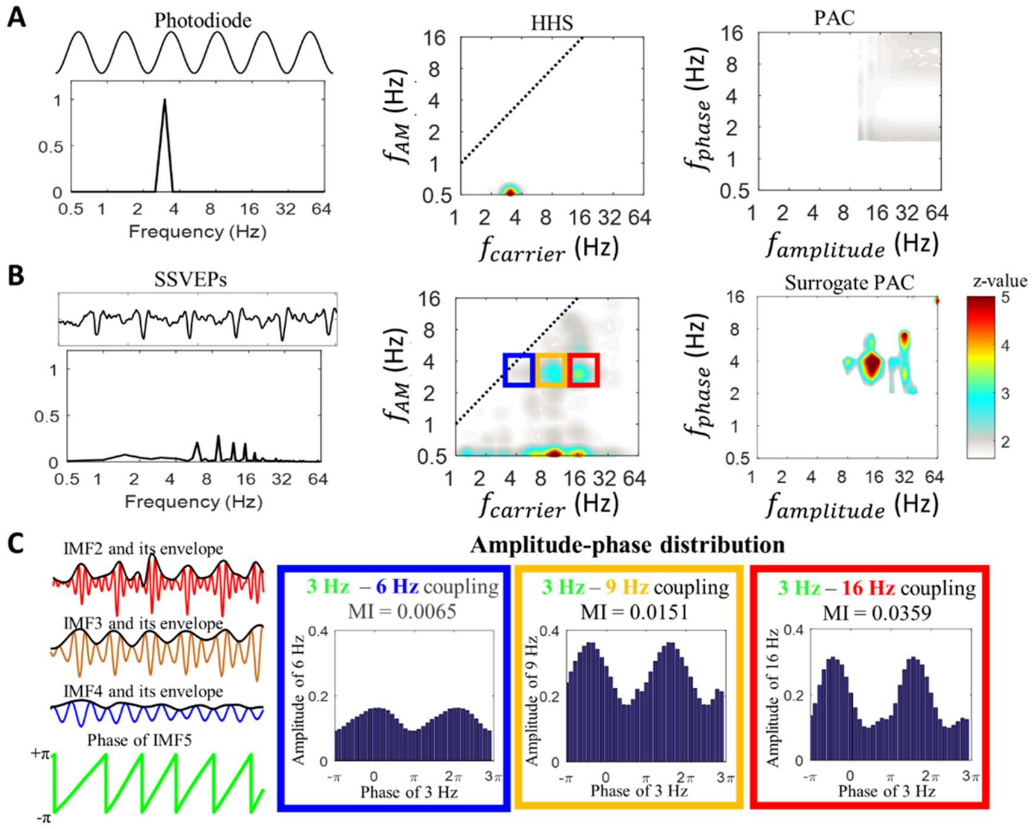

Figure S13. The illustration of measuring the PAC value using adaptive filter (IMFs) from a single participant.

A. The photodiode signal of 3 Hz sinusoidal flicker and the outcomes of HHS and PAC analysis.
B. The SSVEP induced by 3 Hz sinusoidal flicker and the outcomes of HHS and PAC analysis. The rectangles outlined with blue, orange and red indicates three amplitude modulations across IMF4, IMF3 and IMF2, respectively.
C. The IMFs decomposed by EMD. The envelopes of IMF2 (16 Hz peak), IMF3 (9 Hz peak) and IMF4 (16 Hz peak) were extracted using cubic interpolation. The instantaneous phase of IMF5(3 Hz peak) were also extracted by using Hilbert transform. The amplitude-phase distributions and MI values between IMF5 phase and the amplitude of high-frequency IMFs (IMF4, IMF3 and IMF2) are outlined by the blue, orange and red rectangles, respectively.

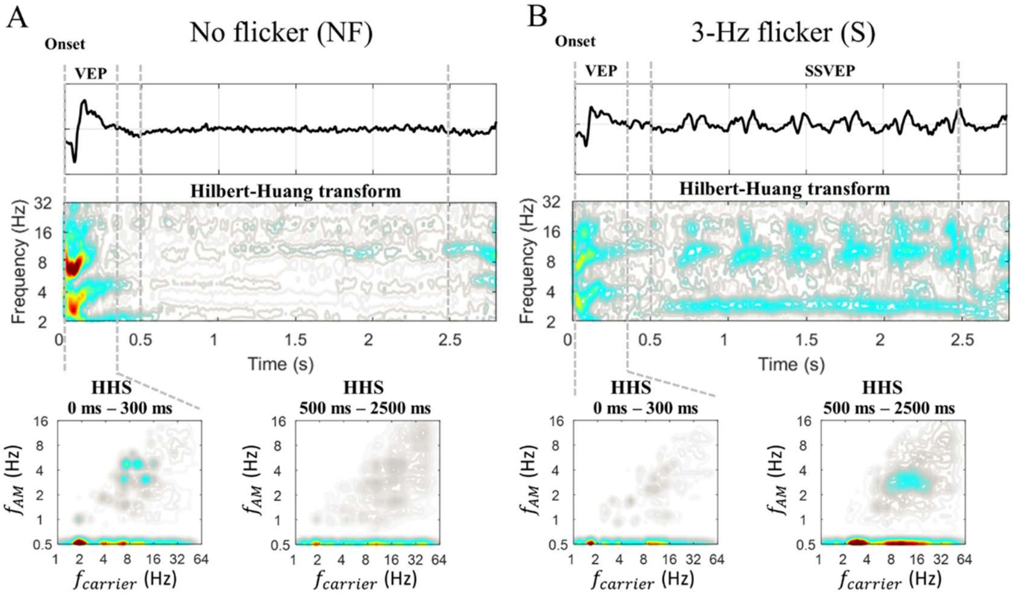

Figure S14. The grand average HHS of the first transient VEP in No flicker (A) and 3 Hz flicker (B), averaged across subjects.
